# Spermidine treatment limits the development of the fungus in flax shoots by suppressing polyamine metabolism and balanced defence reactions, thus increasing flax resistance to fusariosis

**DOI:** 10.3389/fpls.2025.1561203

**Published:** 2025-03-25

**Authors:** Beata Augustyniak, Wioleta Wojtasik, Agnieszka Sawuła, Marta Burgberger, Anna Kulma

**Affiliations:** Faculty of Biotechnology, University of Wrocław, Wroclaw, Poland

**Keywords:** polyamines, spermidine, flax, fusarium oxysporum, plant infection

## Abstract

**Introduction:**

Flax (*Linum usitatissimum*) is an important industrial crop in temperate regions, but fungal diseases, especially those caused by *Fusarium oxysporum* sp. *lini*, pose a serious risk. These infections can lead to major crop losses, reducing interest in flax cultivation.

**Methods:**

This study investigated the effects of exogenous spermidine (Spd) on the interactions between flax and *Fusarium oxysporum* sp. *lini*. Flax plants treated with either 10 mM or 100 mM Spd were monitored for changes in polyamine levels, gene expression, and hydrogen peroxide (H_2_O_2_) content following infection.

**Results and discussion:**

Notably, plants treated with 10 mM Spd showed enhanced resistance, exhibiting better phenotypic health and lower fungal murein levels, especially in shoots. Chitinase expression in these plants remained similar to or lower than control levels, suggesting minimal additional defence activation was required. Additionally, a marked ROS burst occurred two days post-infection, followed by redox balance restoration, indicating a controlled defence response. These results suggest that moderate Spd treatment improves flax resilience against fusarium wilt while avoiding excessive defence activation, highlighting Spd’s potential for sustainable crop protection strategies.

## 1 Introduction

Flax (*Linum usitatissimum*) is a widely cultivated crop in temperate regions, valued for its two primary products: fiber and oil (Kiryluk and Kostecka, 2020). Flax fibers are utilized in textiles and selected biomedical applications, while flaxseed oil, rich in unsaturated fatty acids, is important in the food and chemical industries (Czemplik et al., 2011). By-products such as shives and seedcake enhance its potential for zero-waste utilization (Zuk et al., 2015). Despite its economic importance, flax cultivation faces challenges from diseases like fusarium wilt caused by *Fusarium oxysporum* sp. *lini* (Foln). This pathogen infects plants through the root system, subsequently colonizing the vascular tissues, which disrupts water and nutrient transport. As a result, affected plants exhibit symptoms such as wilting, stunted growth, and eventual death (Pietro et al., 2003). Foln not only reduces crop yields but also compromises fiber and seed quality, amplifying economic losses for flax producers (Olivain et al., 2003). The high adaptability and persistence of Foln in soil make it particularly challenging to control using conventional agricultural practices. Consequently, there is an increasing emphasis on exploring innovative strategies to enhance plant resilience and develop sustainable methods for disease management (Usman et al., 2023).

**Figure 1:**
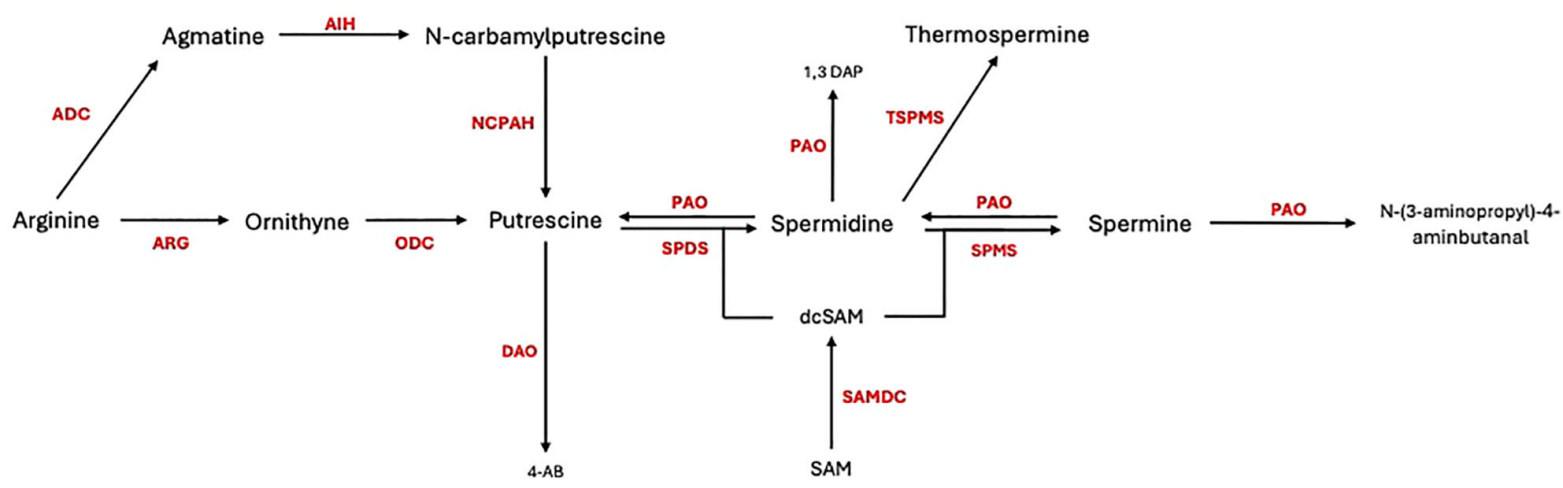
Polyamine biosynthesis and catabolism in plant cells. ADC, arginine decarboxylase; ARG, arginase; AIH, agmatine iminohydrolase; ODC, ornithine decarboxylase; NCPAH, N-carbamoylputrescine amidohydrolase; SPDS, spermidine synthase; SPMS, spermine synthase; DAO, diamine oxidase; PAO, polyamine oxidase; TSPMS, thermospermine synthase; SAMDC, S-adenosylmethionine decarboxylase.

Polyamines (PAs) have garnered significant attention as potential agents in enhancing plant resistance to pathogens. PAs, such as putrescine (Put), spermidine (Spd), spermine (Spm), thermospermine (Tspm), and cadaverine (Cad), are low-molecular-weight polycations present in all living organisms (Mustafavi et al., 2018). These compounds exist in both free and conjugated forms and interact with macromolecules like proteins, nucleic acids, and cell wall components (Chen et al., 2019; Pal et al., 2021). The biosynthesis of PAs in plants begins with the production of putrescine (**Figure 1**). This can occur via two main pathways: [1] arginine is decarboxylated by arginine decarboxylase (ADC, EC 4.1.1.19) to produce agmatine, which is then converted into putrescine through the actions of agmatine iminohydrolase (AIH, EC 3.5.3.12) and N-carbamoylputrescine amidohydrolase (NCPAH, EC 3.5.1.53), or [2] arginine is converted to ornithine by arginase (ARG, E.C. 3.5.3.1), ornithine then is converted into putrescine by ornithine decarboxylase (ODC, EC 4.1.1.17) (Docimo et al., 2012). Putrescine serves as the precursor for higher polyamines, including spermidine and spermine, which are synthesized through successive reactions catalysed by spermidine synthase (SPDS, EC 2.5.1.16) and spermine synthase (SPMS, EC 2.5.1.22), using aminopropyl groups derived from the decarboxylation of S-adenosylmethionine (SAM) by S-adenosylmethionine decarboxylase (SAMDC, EC 4.1.1.50). The catabolism of PAs occurs through oxidation by copper-containing amine oxidases (DAOs, E.C. 1.4.3.22) and flavin-containing polyamine oxidases (PAOs, E.C. 1.5.3.14), leading to the production of hydrogen peroxide, ammonia, and 4-aminobutanal. These metabolic processes are tightly regulated, ensuring that PAs levels are maintained at an optimal range for cellular function (Gonzalez et al., 2021).

Polyamines play a complex role in plant-pathogen interactions, influencing immune responses through both direct and indirect mechanisms. One such mechanism involves PAs acting as elicitors of plant defence, where they activate MAPK pathways, leading to the upregulation of defence-related genes and the initiation of hypersensitive responses, which localize and limit pathogen spread by inducing cell death (Hussain et al., 2011). Additionally, PAs are involved in maintaining redox homeostasis. During PA catabolism, reactive oxygen species (ROS) like hydrogen peroxide are produced, which can directly inhibit pathogen growth and trigger further defence signalling, including cell wall reinforcement through lignification. PAs also interact with other key signalling molecules, such as nitric oxide (NO), salicylic acid, and abscisic acid, enhancing the plant’s ability to withstand pathogen attack. In response to fungal infections, elevated levels of PAs, particularly putrescine and spermidine, have been observed in infected plant tissues, suggesting that PAs play a crucial role in modulating plant susceptibility or resistance to pathogens (Walters, 2003; Liu and Alcazar, 2021).

The influence of PAs in plant-fungal interactions varies depending on factors such as polyamine type, concentration, and host species. For instance, elevated levels of putrescine and spermidine have been reported in wheat leaves infected by *Pyricularia oryzae*, suggesting that their accumulation may impact plant vulnerability to the pathogen (Aucique-Pérez et al., 2020). Exogenous application of specific PAs can either bolster or impair plant resistance, depending on the context. Spermine treatment in *Solanum lycopersicum* and *Arabidopsis thaliana* has been shown to enhance resistance to *Botrytis cinerea* by inducing systemic acquired resistance (SAR) and increasing ROS production (Seifi et al., 2019). Furthermore, research on maize infected by *Aspergillus flavus* has revealed that resistant varieties exhibit higher basal expression of genes involved in PA metabolism, which likely contributes to their enhanced defence capabilities (Majumdar et al., 2019). These findings highlight the multifaceted role of PAs in shaping the outcomes of plant-pathogen interactions.

The objective of this study was to investigate how spermidine influences the interaction between flax and *Fusarium oxysporum*. To address this, we decided to elevate the spermidine content in flax by adding it to the medium, testing whether this exogenous polyamine could be absorbed by the plant roots and subsequently transported to the shoots. Next, we would investigate how *Fusarium oxysporum* infection alters polyamine metabolism in flax. This approach would ultimately allow us to explain the effect of increased spermidine levels on flax infection caused by Foln. In these plants, we would analyse the content of other polyamines, the transcript levels of polyamine metabolism genes, and the content of H_2_O_2_. This study aims to deepen our understanding of how polyamine metabolism contributes to the plant’s defence mechanisms. Understanding these interactions may help develop strategies to enhance flax resistance to fusarium wilt and improve sustainable flax cultivation practices.

## 2 Materials and methods

### 2.1 Plant material and growing conditions

Flax seeds (*Linum usitatissimum* L., cv. Nike) were sourced from the Flax and Hemp Collection of the Institute of Natural Fibers and Medicinal Plants - National Research Centre (INF&MP-NRC) in Poland. The pathogenic strain *Fusarium oxysporum* f. sp. *lini* (Bolley) Snyder et Hansen (ATCC MYA-1201) was obtained from the American Type Culture Collection (ATCC) in the USA.

The flax seeds were sterilized by immersing them in 50% PPM (Plant Cell Technology, UK) for 10 minutes and then transferred to sterile jars containing 45 ml of Murashige and Skoog medium (MS medium; Sigma-Aldrich), adjusted to pH 5.8, with 10 g of vermiculite. The flax plants were grown in a controlled environment chamber under a 16-hour light (21°C) and 8-hour dark (16°C) photoperiod.

A 1 M spermidine stock solution (Spermidine trihydrochloride; Sigma-Aldrich) was prepared by dissolving the appropriate amount of spermidine in distilled water. The solution was sterilized using a 0.22 µm syringe filter in a laminar flow hood. The final concentrations of 10 mM and 100 mM spermidine were achieved by diluting the stock solution with sterile water. These concentrations were chosen based on the treatment plan and plant growth conditions. Since the spermidine was applied to the solid substrate once, the actual concentration available to the plants would be lower, in line with values reported in the literature (Szalai et al., 2017; van Rensburg et al., 2021).

**Figure 2:**
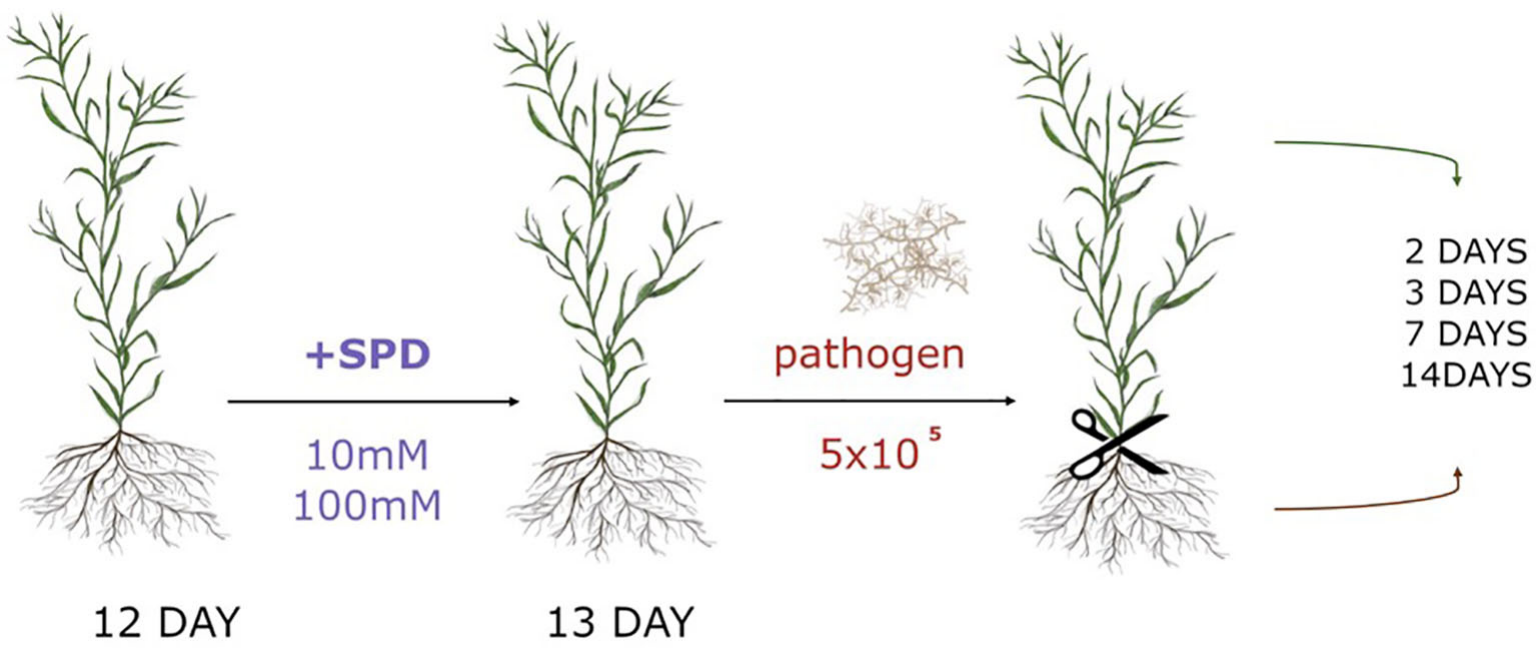
Experimental scheme of spermidine (Spd) treatment and *F.oxysporum* (pathogen) application with 5x10^5^ spores amount (10^6^ spores/ml).

Twelve-day-old *in vitro*-grown flax plants were treated with 5 ml of either 10 mM or 100 mM spermidine solution by watering the substrate. After 24 hours, the plants were exposed to the pathogenic fungus *F. oxysporum*. The fungus was cultured on potato dextrose agar (PDA, IBI Scientific) at 28°C, and spore viability was assessed by determining the percentage of germinated spores. Spores, including both macro- and microconidia, were harvested by flooding the culture plate with 15 ml of sterile water. The spore concentration was adjusted to 10^6^ spores/ml using a hemocytometer. For inoculation, 0.5 ml of the spore suspension (10^6^ spores/ml) or 0.5 ml of sterile water (control) was added to the culture medium of both untreated and spermidine-treated flax plants (**Figure 2**).

Flax plants treated with spermidine, untreated controls, flax infected with *F. oxysporum*, and flax treated with spermidine and infected with *F. oxysporum* were harvested at 2, 3, 7, and 14 days post-infection. Roots and shoots were collected separately. Each experimental group at each time point consisted of three jars, each containing 16 plants. The plants from each jar were pooled as a single sample, ground in liquid nitrogen, and weighed for subsequent analyses.

### 2.2 Gene expression analysis

The mRNA levels of the analysed genes were measured using real-time PCR. Total RNA from the roots was extracted using the Spectrum™ Plant Total RNA Kit (Sigma-Aldrich) according to the manufacturer’s instructions, while RNA from the shoots was isolated with RNA Extracol (EURx), following the provided protocol. RNA integrity was verified through 1% (w/v) gel electrophoresis, and the RNA concentration was quantified spectrophotometrically. Any remaining DNA was removed with DNase I (Invitrogen) treatment. The purified RNA was then used as a template for cDNA synthesis, utilizing the High-Capacity cDNA Reverse Transcription Kit (Applied Biosystems).

Real-time PCR was conducted using SG/ROX qPCR Master Mix (EURx) on an Applied Biosystems StepOnePlus Real-Time PCR System. The reactions followed the kit manufacturer’s instructions, with an annealing temperature of 57°C. The specificity of the primers at this temperature was confirmed by analysing the melting curves of the products. Primer sequences used for the reactions are shown in **Table 1**. Each reaction was performed in triplicate. The actin gene was used as a reference for normalization. Transcript level changes were calculated relative to the reference gene.

**Table 1:** Primer sequences used for the real-time PCR reactions.

Gene	Forward Primer	Reverse primer
*ACT*	5′ CCGGTGTTATGGTTGGAAT 3′	5′ TGTAGAAAGTGTGATGC CAAA 3′
*CHIT*	5’ CATCCAATGAATGGCCTT 3,	5’ GGCTGTTCGGAATGATATCTC 3’
*ADC*	5′ GAGGAGCTTGATTTGGTGA 3′	5′ CACGCTGAGAATCTGAGTT 3′
*AIH*	5′ GTCTACTGCAACTGATGCTAA 3′	5′ AGAAGCAGCAAGTCTTGT 3′
*NCPAH*	5′ AAGCACAAAGGGAGGAC 3′	5′ CTATGGAATTGTAATGAGCAT TGTT 3′
*ARG*	5′ CTGATGTTGGTGATGTCCC 3′	5′ CCTCCAAGTTTCTCAGATAC AG 3′
*ODC*	5′ CTGGATGAGTGTTTCTCAGG 3′	5′ GCAATTCATCGACCCGTA 3′
*SPDS*	5′ AAAGCAGTCCTGGTAGTTG 3′	5′ CGTGGATCTTCGAATCCG 3′
*SPMS*	5′ GGCTATTCCTTCCTGTTGTC 3′	5′ GGCCACATAGGATTGTTAA AGTA 3′
*DAO*	5′ CAAAGTCGGCATGATCG 3′	5′ GGGTTTACGTTGACCAGAG 3′
*PAO*	5′ ATGGGAGGACGTTTGTAG 3′	5′ GCCAGAACACATTGTCGAA 3′

ACT, actin; CHIT, chitinase; ADC, arginine decarboxylase; AIH, agmatine iminohydrolase; NCPAH, N-carbamoylputrescine amidohydrolase; ARG, arginase; ODC, ornithine decarboxylase; SPDS, spermidine synthase; SPMS, spermine synthase; DAO, diamine oxidase; PAO, polyamine oxidaseõ.

### 2.3 Polyamine extraction and analysis by UPLC

The isolation and fractionation of polyamines was performed using a modified version of the method described by Wojtasik et al (2015). Briefly, 100 mg of ground flax roots or shoots were extracted with 1ml of 4% (w/v) perchloric acid (PCA; VWR Chemicals) for 1h at 4°C with shaking. After centrifugation (14,000 rpm, 20 min, 4°C), the supernatant was preserved, and the pellet was resuspended in 0.5 ml of 4% PCA. The supernatant contains free polyamines and polyamines conjugated to phenolic acids, and the pellet contains insoluble polyamines bound to cell wall components.

In order to convert the conjugated and bound forms into a free form, 0.5 ml of 6 N HCl (Chempur) were added to 0.5 ml of the supernatant and suspended pellet and hydrolyzed at 110°C for 20h. After drying in a block heater at 100°C, the residues were re-dissolved in 0.5 ml of 4% PCA.

Subsequently, polyamine dansylation was performed to facilitate identification: 0.2 ml aliquots were added to 0.2 ml of saturated sodium carbonate (Merck, Supelco) and 0.4 ml of dansyl chloride (5 mg/ml in acetone). After brief vortexing, the mixture was incubated in darkness at 60°C for 1h. Excess dansyl reagent was removed by adding 0.2 ml of 150 mg/ml proline. Dansylated polyamines were extracted by 0.5 ml of toluene (VWR Chemicals), dried under nitrogen flow and re-suspended in 0.2 ml of acetonitrile (VWR Chemicals), and finally 4 μl were injected on the column.

The samples were analysed using a Waters Acquity UPLC system with a 2996 PDA detector on an Acquity UPLC HSS T3 2,1 x 100 mm 1.8 μm column. The mobile phase was A = water and B = acetonitrile, in a gradient flow: initial: 40% A/60% B; 1.17 min: 40% A/60% B; 1.65 min: 30% A/70% B; 4.0 min: 25% A/75% B; 7.57 min: 0% A/100% B; 8.00 min: 0% A/100% B; 8.5: 40% A/60% B; 11.5 min: 40% A/60% B with a 0.4 ml/min flow rate. The peak integration was done at 366 nm.

The results were standardized with mixtures of dansylated polyamine standards (putrescine, spermidine, spermine). Conjugated polyamine content was calculated by subtracting free polyamine content from the total acid-soluble polyamine content. For most of the analysed samples, the amount of soluble conjugates was below the detection limit. Therefore, the data were ultimately not included in further analysis.

### 2.4 Monitoring the progress of the infection

Phenotypic changes illustrating the symptoms of the disease (yellowing of leaves, brown spots on the leaves, wilting) were assessed visually and photographed. Phenotypic changes in the studied plants, 3 jars from each experimental group were left for regular monitoring.

Genomic fungal DNA was isolated from the roots and shoots of infected plants using the CTAB method. In brief, approximately 100 mg of ground plant tissue was lysed in 700 µl of CTAB buffer (2% CTAB, 100 mM Tris-HCl pH 8.0, 20 mM EDTA, 1.4 M NaCl, 1% β-mercaptoethanol), with polyvinylpolypyrrolidone (PVPP) added to remove polyphenols. The mixture was incubated at 65°C for 30 minutes with occasional inversion. After lysis, the mixture was centrifuged at 14,000 rpm for 10 minutes at room temperature. The supernatant was transferred to a new tube, while the pellet was discarded. To eliminate proteins and polysaccharides, 450 µl of a chloroform:isoamyl alcohol (24:1) mixture was added to the supernatant, gently mixed, and centrifuged again at 14,000 rpm for 8 minutes. The upper aqueous phase was carefully removed, avoiding the interphase. This extraction step was repeated to ensure the purity of the DNA. DNA was precipitated by adding an equal volume of isopropanol and 30 µl of 3M sodium acetate (pH 5.2), followed by incubation at -20°C for 15 minutes and centrifugation at maximum speed for 10 minutes. The DNA pellet was washed with 500 µl of 70% ethanol, gently vortexed, and centrifuged at 7,500 rpm for 5 minutes. After discarding the supernatant, the pellet was dried at 56°C for 5 minutes. The DNA was then resuspended in 40 µl of RNase-treated water (0.5 µg/µl) and incubated at 65°C for 10 minutes to remove residual RNA. DNA integrity was assessed by 1.0% (w/v) agarose gel electrophoresis, and DNA concentration was measured spectrophotometrically.

The extracted DNA (100 ng/µl) was used as a template for real-time PCR targeting a 150 bp fragment of the fungal murein transglycosylase gene (primers: Fwd: TCTCAACGGTGTCGAGTCTAA, Rev: CACCCTGGTTGCAGATAAT), with the actin gene serving as a reference. Real-time PCR reactions were conducted using SG/ROX qPCR Master Mix (EURx) on an Applied Biosystems StepOnePlus Real-Time PCR System. The reaction conditions followed the manufacturer’s protocol, with the annealing temperature set at 57°C.

### 2.5 H_2_O_2_ content determination

Plant material was ground in liquid nitrogen using a mortar and pestle and 50 mg of the obtained powder was used for processing. The ground tissue was suspended in 200 µL of 20 mM phosphate buffer pH 6.5 and centrifuged (10 min, 4°C, 10,000× g). The supernatant was transferred to a new Eppendorf tube and 50 µL was used for H_2_O_2_ content determination. The assay was performed using Amplex™ Red Hydrogen Peroxide/Peroxidase Assay Kit (Life Technologies) according to the producer’s instructions. Varioskan Flash (Thermo Scientific) was used for samples measurement.

### 2.6 Statistical analysis

Each experimental group comprised three jars, with each jar containing 16 plants. The plants were collected and treated as a single pool. The results of the experiments were presented as the mean of the three repetitions, accompanied by the corresponding standard deviation. Statistical analyses were performed using Prism Graphpad 10 software. The significance of the differences between studied groups was determined using two-way ANOVA with Tuckey *post hoc* test.

## 3 Results

### 3.1 The impact of *Fusarium oxysporum* infection on flax

#### 3.1.1 Progression of the infection with *Fusarium oxysporum* in flax

**Figure 3:**
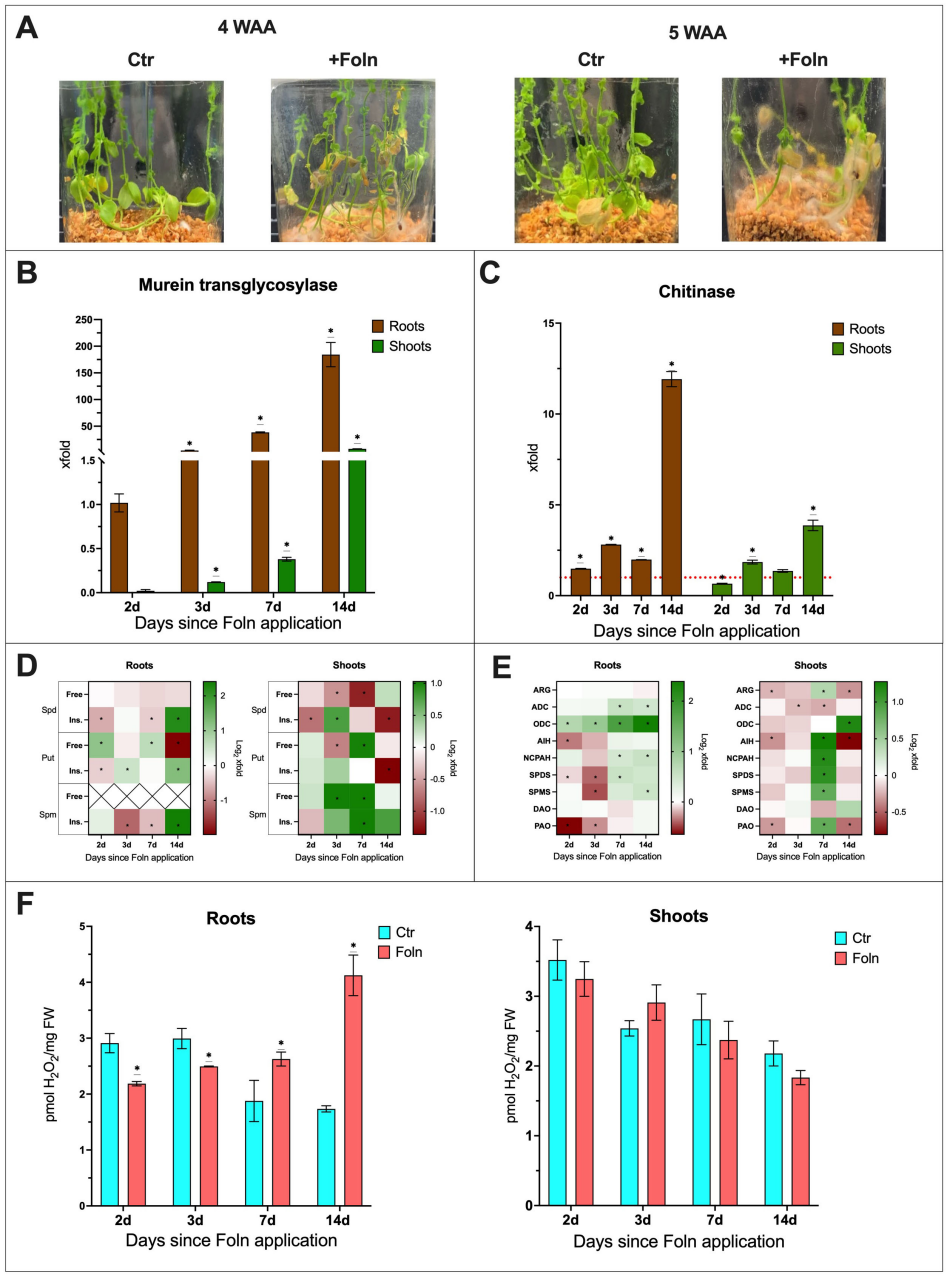
The effect of *F oxysporum* infection on flax plants. **(A)** Phenotypic changes in plants infected with *F oxysporum.* (WAA, weeks after application; Ctr, control). **(B)** Relative quantity of fungal *murein transglycosylase* gene in roots and shoots of plants after Foln application. The data presented were obtained from real-time PCR analysis. Flax *actin* served as the reference gene, and the relative quantity of the *murein transglycosylase* gene was normalized to infected roots 2 days after Foln application. Bars represent the mean ± SD from three replicates. The significance of differences between groups was determined using two-way ANOVA followed by Tukey’s *post hoc* test (*P < 0.05 for comparison to 2-day control). **(C)** Transcript level of *chitinase* gene in flax roots and shoots after Foln application. The data were obtained from real-time RT-PCR analysis. *Actin* was used as a reference gene and the transcript levels were normalized to the control noninfected plants (marked as red dot line). Bars represent the mean ± SD from three replicates. The significance of differences between groups was determined using two-way ANOVA followed by Tukey’s *post hoc* test (*P < 0.05 for comparison to control from the same time point as the sample). **(D)** Polyamine (SPD, spermidine; PUT, putrescine; SPM, spermine) content in flax roots and shoots after *F oxysporum* application. Heat map box represent the mean from three replicates. The significance of differences between groups was determined using two-way ANOVA followed by Tukey’s *post hoc* test (*P < 0.05 for comparison to control from the same time point as the sample). **(E)** Transcript level of polyamine metabolism genes in flax roots and shoots after Foln application. The data were obtained from real-time RT-PCR analysis. *Actin* was used as a reference gene and the transcript levels were normalized to the untreated control plant. Heat map box represent the mean from three measurements. The significance of the differences was determined using two-way ANOVA with Tuckey *post hoc* test (*P<0,05) (ARG, arginase; ADC, arginine decarboxylase; ODC, ornithine decarboxylase; AIH, agmatine iminohydrolase; NCPAH, N-carbamoylputrescine amidohydrolase; SPDS, spermidine synthase; SPMS, spermine synthase; DAO, diamine oxidase; PAO, polyamine oxidase). **(F)** H_2_O_2_ content in flax roots and shoots after Foln application. Bars represent the mean ± SD from three replicates. The significance of differences between groups was determined using two-way ANOVA followed by Tukey’s *post hoc* test (*P < 0.05 for comparison to control from the same time point as the sample).

Phenotypic observations showed that starting from the fourth week after pathogen application, plants displayed signs of infection, such as leaf yellowing and wilting, which were not observed in the non-infected control plants (**Figure 3A**). To describe the infection progression, the increase in fungal DNA levels in the plants was tracked through analysis of the fungal *murein transglycosylase* gene (**Figure 3B**). Plant responses to the infection were represented by changes in the transcript levels of the *chitinase* gene, a marker of pathogenesis-related (PR) responses. (**Figure 3C**). In infected plants, an increase in the relative amount of the *murein transglycosylase* gene was observed. In the roots, there was a 4,5-fold increase in the *murein transglycosylase* gene level by day 3 after Foln application, a 38-fold increase by day 7 and a 184-fold increase by day 14 compared to day 2. In shoots, the *murein transglycosylase* gene level increased from 0,02 on day 2 to 0,1 on day 3, 0,4 on day 7,5 on day 14 compared to roots day 2. Notably, a much lower level of fungal *murein transglycosylase* gene was observed in the shoots compared to the roots.

In the roots of infected plants, an increase in *CHIT* transcript levels was observed from day 2 post Foln application compared to non-infected plants (1,5-fold increase on day 2, 2,8-fold increase on day 3, 2-fold increase on day 7 and 12-fold increase on day 14). In the shoots of infected plants, there was initially a decrease in *CHIT* mRNA levels, with a 35% reduction on day 2. However, at subsequent time points, a similar increase in *CHIT* transcript levels was observed as in the roots, with an 1,8-fold increase on day 2 and a 3,8-fold increase on day 14.

#### 3.1.2 The effect of infection on polyamine content

To assess the impact of infection on the levels of PAs in the roots and shoots of flax, the following data were described as the fold change in plants infected with *F. oxysporum* compared to uninfected controls (**Figure 3D**). The absolute levels are presented in supplementary files (**Supplementary Figure S1**).

In the roots of infected plants, the level of free spermidine remained unchanged throughout the course of infection. A decrease in the amount of insoluble conjugates was observed 2 days (35%) and 7 days (25%) after Foln application. However, 14 days after Foln application, there was a 4.6-fold increase in this spermidine fraction in the roots of infected plants compared to the uninfected control. In the shoots of infected plants, a reduction in free spermidine levels was observed (33% at 3 days after Foln application and 56% at 7 days after Foln application). Additionally, in the shoots, a 40% decrease in the insoluble spermidine conjugate fraction was observed 2 days after Foln application. By day 3, the level of this fraction had increased 1.7-fold in the infected shoots. However, by day 14, the level had dropped again by 60%.

In the roots of infected plants, an increase in free putrescine levels was observed 2 days (2.2-fold) and 7 days (1.6-fold) after Foln application. However, 14 days after Foln application, there was a 74% decrease in this fraction of putrescine compared to the roots of uninfected plants. For insoluble putrescine conjugates, a decrease was observed at the first time point studied, with a 23% reduction 2 days after Foln application. 3 days after Foln application, the level of insoluble putrescine conjugates increased 1.4-fold, and 14 days after pathogen application, it increased 2.4-fold. In the shoots of infected plants, free putrescine, like spermidine, initially showed no changes. However, 3 days after Foln application, a 37% decrease in free putrescine levels was observed in infected shoots. 7 days after Foln application, the level of free putrescine increased 2-fold. The level of insoluble putrescine conjugates did not show significant changes in the early stages of infection in the shoots of infected plants. However, by day 14 after Foln application, there was a 61% decrease in insoluble putrescine conjugates.

In the roots of infected plants, a decrease in insoluble spermine conjugates was observed on days 3 and 7 after Foln application (by 55% and 30%, respectively). However, 14 days after Foln application, there was a 5.3-fold increase in this spermine fraction in the examined roots. In the infected shoots, a 2-fold increase in free spermine levels was observed on both days 3 and 7 after Foln application. Insoluble spermine conjugates also exhibited an upward trend, with a 2-fold increase 7 days after Foln application.

#### 3.1.3 The effect of infection on transcript levels of polyamine metabolism genes

To assess the impact of infection on the levels of polyamine metabolism genes mRNA in the roots and shoots of flax, the following data were described as the fold change in plants infected with *F. oxysporum* compared to uninfected controls (**Figure 3E**; **Supplementary Figure S2**).

In the roots of infected plants, the transcript level of the *ARG* gene did not show significant changes. However, 2 days after Foln application, a 20% decrease in *AIH* transcript levels was observed. The mRNA levels of the *ADC* and *NCPAH* genes remained relatively stable during the early stages of infection, but after 7 and 14 days of Foln application, the mRNA levels of both genes increased 1.5-fold. In the roots of infected plants, a continuous increase in the transcript level of the *ODC* gene was observed, starting 2 days after Foln application with a 1.7-fold increase, followed by a 2-fold increase at day 3, a 3.5-fold increase at day 7, and reaching a 5.2-fold increase at day 14.

In the shoots of infected plants, a slight decrease in the transcript levels of *ARG*, *ADC*, and *AIH* genes was observed during the early stages of infection (15% decrease in *ARG* 2 days after Foln application; 14% decrease in *ADC* 3 days after Foln application; 22% decrease in *AIH* on day 2 after Foln application). 7 days after Foln application, the transcript levels of *ARG* (1.3-fold), *AIH* (2.4-fold), and *NCPAH* (2.2-fold) increased, while the mRNA level of the *ADC* gene decreased by 17%. 14 days after Foln application, there was a 21% decrease in *ARG* mRNA levels and a 43% decrease in *AIH* mRNA levels.

In the shoots of infected plants, no changes in *ODC* mRNA levels were observed at the early time points studied. However, by day 14 after pathogen application, the transcript level of this gene increased 2.8-fold in infected shoots.

3 days after Foln application, the mRNA levels of the *SPDS* and *SPMS* genes in the roots of infected plants decreased by 24% and 27%, respectively. By day 7 after Foln application, there was a 1.5-fold increase in *SPDS* transcript levels. By day 14, the *SPMS* mRNA levels also showed a 1.5-fold increase. In the shoots of infected plants, 7 days after pathogen application, a 2.2-fold increase in SPDS transcript levels and a 1.8-fold increase in SPMS transcript levels were observed. No significant changes in shoots in the mRNA levels of these genes were detected at other time points.

In both the roots and shoots of infected plants, no significant changes were observed in the transcript levels of the *DAO* gene. However, 2 days after pathogen application, the mRNA level of the *PAO* gene in the roots of infected plants decreased by 36%, and by day 3, it remained 20% lower. Similarly, in the shoots of infected plants, the *PAO* mRNA level was reduced by 20% at day 2 after pathogen application. By day 7 after Foln application, the *PAO* transcript level increased 1.8-fold, but by day 14, it dropped by 37%.

#### 3.1.4 The effect of infection on H_2_O_2_ level

The levels of hydrogen peroxide (H_2_O_2_) were also measured in the studied plants. In the roots of infected plants, an initial significant decrease in H2O2 levels was observed, with a 25% reduction on the 2nd and 3rd days after Foln application. However, 7 days after Foln application, the H2O2 level increased 1.65-fold. By day 14, the H2O2 level had risen by 1.9-fold. In the shoots of infected plants, no significant changes were observed. (**Figure 3F**).

### 3.2 The effect of spermidine treatment on flax

**Figure 4:**
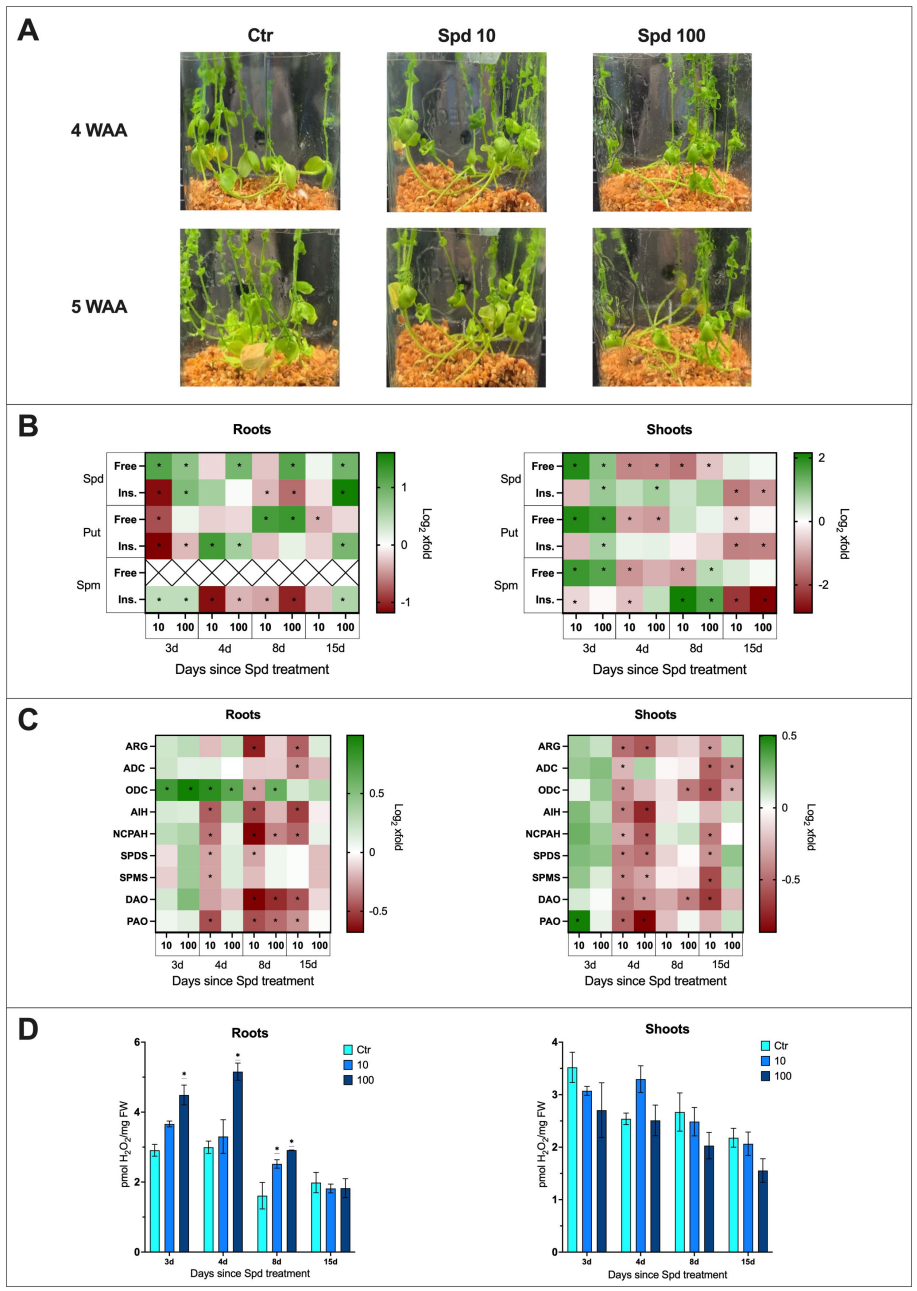
The effect of spermidine treatment on flax plants. **(A)** Phenotypic changes in plants treated with spermidine [10 mM and 100 mM] (WAA, weeks after application; Ctr, control). **(B)** Polyamine (SPD, spermidine; PUT, putrescine; SPM, spermine) content in flax roots and shoots after spermidine treatment. Heat map box represent the mean from three replicates. The significance of differences between groups was determined using two-way ANOVA followed by Tukey’s *post hoc* test (*P < 0.05 for comparison to control from the same time point as the sample). **(C)** Transcript level of polyamine metabolism genes in flax roots and shoots after Spd treatment [mM]. The data were obtained from real-time RT-PCR analysis. *Actin* was used as a reference gene and the transcript levels were normalized to the untreated control plant. Heat map box represent the mean from three measurements. The significance of the differences was determined using two-way ANOVA with Tuckey *post hoc* test (*P<0,05) (ARG, arginase; ADC, arginine decarboxylase; ODC, ornithine decarboxylase; AIH, agmatine iminohydrolase; NCPAH, N-carbamoylputrescine amidohydrolase; SPDS, spermidine synthase; SPMS, spermine synthase; DAO, diamine oxidase; PAO, polyamine oxidase). **(D)** H_2_O_2_ content in flax roots and shoots after Spd treatment [mM]. Bars represent the mean ± SD from three replicates. The significance of differences between groups was determined using two-way ANOVA followed by Tukey’s *post hoc* test (*P < 0.05 for comparison to control from the same time point as the sample).

The phenotypic observation of plants treated solely with spermidine revealed that none of the tested concentrations had a significant effect on the phenotype of plants compared to untreated plants (**Figure 4A**).

#### 3.2.1 The effect of spermidine treatment on polyamine levels

It was important to determine if plants take up spermidine added to the medium. After confirming an increase in spermidine levels in treated plants, the impact on the levels of other PAs was analyzed. The following data (**Figure 4B**) were described as the fold change in Spd-treated plants compared to untreated control plants. The absolute levels are presented in supplementary files (**Supplementary Figure S3**).

In the roots of plants treated with Spd, a 2.2-fold increase in free spermidine levels was observed 3 days after treatment with 10 mM Spd, and a 1.7-fold increase after treatment with 100 mM Spd. Over time, the free spermidine levels in plants treated with 10 mM Spd no longer differed from the control. However, in plants treated with 100 mM Spd, elevated levels of free spermidine persisted: 1.9-fold higher at day 4, 2.2-fold at day 8, and 2-fold at day 15 after treatment. Additionally, 3 days after treatment with 100 mM Spd, there was a 1.8-fold increase in the levels of insoluble spermidine conjugates, while treatment with 10 mM Spd led to a 54% decrease in this fraction. By day 8, a decrease in the levels of insoluble spermidine conjugates was observed for both concentrations (21% for 10 mM and 37% for 100 mM). However, 15 days after treatment with 100 mM Spd, a 3.1-fold increase in insoluble spermidine conjugates was detected in the treated roots.

In the shoots of plants treated with Spd, 3 days after treatment with 10 mM Spd, a 4-fold increase in free spermidine levels was observed, while treatment with 100 mM Spd resulted in a 2-fold increase. However, by the following day, a 60% decrease in free Spd levels was noted for both concentrations. This reduced level of free Spd persisted 8 days after treatment, with a 66% decrease following 10 mM Spd treatment and a 37% decrease after 100 mM Spd treatment. By day 15, the levels of free spermidine for both concentrations were comparable to those in untreated shoots. In the shoots of plants treated with 100 mM Spd, a 1.9-fold increase in insoluble spermidine conjugates was observed 3 days after treatment, which remained at the same level 4 days after treatment. By day 15, a significant decrease in the levels of insoluble spermidine conjugates was observed, with a 65% reduction after 10 mM Spd treatment and a 56% reduction following 100 mM Spd treatment.

In the roots treated with 10 mM Spd, a 43% decrease in free Put levels was observed 3 days after treatment. The higher concentration did not cause any significant changes compared to the control in the early stages after treatment. By day 8, there was a 2.4-fold increase in free Put levels after treatment with 10 mM Spd and a 2.5-fold increase following 100 mM Spd treatment. However, by day 15, a 22% decrease in free Put levels was again observed in the roots treated with 10 mM Spd. After Spd treatment, the levels of insoluble Put conjugates in the roots initially dropped by 55% following 10 mM Spd treatment and by 20% following 100 mM Spd treatment. However, 4 days after treatment, there was a 2.4-fold increase in insoluble Put conjugates after 10 mM Spd treatment and a 1.5-fold increase after 100 mM Spd treatment. By day 15, a 1.9-fold increase in insoluble Put conjugates was only observed after treatment with 100 mM Spd.

In the shoots of plants treated with Spd, a 4-fold increase in free Put levels was observed 3 days after treatment with 10 mM Spd, and a 3.4-fold increase after treatment with 100 mM Spd. However, by day 4, the levels of free Put decreased by 50% following treatment with 10 mM Spd and by 55% following 100 mM Spd. By day 15, a 30% decrease in free Put levels was still observed in the shoots treated with 10 mM Spd. Three days after treatment with 100 mM Spd, there was a 1.7-fold increase in the levels of insoluble Put conjugates. By day 15, both concentrations of Spd led to a 60% decrease in the levels of insoluble Put conjugates in the treated shoots.

3 days after Spd treatment, a 1.3-fold increase in the levels of insoluble spermine conjugates was observed with both concentrations of Spd. However, by day 4, the levels of insoluble Spm conjugates decreased following treatment with both concentrations (a 54% decrease after 10 mM Spd and a 22% decrease after 100 mM Spd). By day 8, the decline in this fraction of Spm continued in the treated roots (a 26% decrease after 10 mM Spd and a 51% decrease after 100 mM Spd). 15 days after treatment with 10 mM Spd, the levels of insoluble Spm conjugates still showed an 18% decrease.

3 days after Spd treatment, an increase in free spermine was observed in the treated shoots (3.4-fold after 10 mM Spd; 2.9-fold after 100 mM Spd). By day 4, treatment with 10 mM Spd resulted in a 56% decrease in free Spm levels. The decline continued by day 8, with a similar 53% reduction. At this point, however, treatment with 100 mM Spd led to a 1.5-fold increase in free Spm levels in the treated shoots. A decrease in the levels of insoluble Spm conjugates was noted 3 and 4 days after treatment with 10 mM Spd, with reductions of 28% and 40%, respectively. By day 8, there was a significant increase in insoluble Spm conjugates – 4.5-fold after treatment with 10 mM Spd and 3-fold after 100 mM Spd. 15 days after Spd treatment, a sharp decrease in the levels of insoluble Spm conjugates was observed – 79% after 10 mM Spd and 85% after 100 mM Spd.

#### 3.2.2 The effect of spermidine treatment on transcript levels of polyamine metabolism genes

To assess the impact of Spd treatment on the levels of polyamine metabolism genes mRNA in the roots and shoots of flax, the following data were described as the fold change in plants treated with Spd compared to untreated controls (**Figure 4C**; **Supplementary Figures S4**, **S5**).

Treatment with Spd appears to lower the transcript levels of most genes related to polyamine metabolism. In the treated plant roots, an increase in transcript levels was only observed for the *ODC* gene following 10 mM Spd treatment, with a 1.7-fold increase 3 days after treatment and a 1.8-fold increase after 4 days. For 100 mM Spd, there was a 2-fold increase after 3 days, a 1.6-fold increase after 4 days, and a 1.5-fold increase after 8 days. In the treated shoots, a 1.4-fold increase in transcript level was observed only for the *PAO* gene, 3 days after 10 mM Spd treatment.

In general, treatment with 10 mM Spd led to a reduction in the transcript levels of most polyamine metabolism-related genes at 4, 8, and 15 days after treatment. 4 days after treatment with 10 mM Spd, transcript levels in the roots decreased as follows: *AIH* by 26%, *NCPAH* by 23%, *SPDS* by 16%, *SPMS* by 16%, and *PAO* by 28%. By 8 days after treatment, the transcript levels decreased for *ARG* by 34%, *ODC* by 17%, *AIH* by 29%, *NCPAH* by 38%, *SPDS* by 15%, *DAO* by 37%, and *PAO* by 28%. After 15 days, a similar trend continued, with reductions in *ARG* by 26%, *ADC* by 19%, *AIH* by 30%, *NCPAH* by 25%, *DAO* by 28%, and *PAO* by 19% in the treated roots.

Interestingly, treatment with 100 mM Spd caused fewer transcript changes, and only at 8 days post-treatment, with decreases in *NCPAH* by 20%, *DAO* by 33%, and *PAO* by 24%.

In shoots of treated plants, the genes responded similarly. 4 days after treatment with 10 mM Spd, the transcript levels decreased as follows: *ARG* by 29%, *ADC* by 16%, *ODC* by 23%, *AIH* by 27%, *NCPAH* by 19%, *SPDS* by 26%, *SPMS* by 25%, *DAO* by 28%, and *PAO* by 31%. By 8 days post-treatment with 10 mM Spd, no significant changes in transcript levels of the studied genes were observed. However, at 15 days post-treatment, there were once again reductions in transcript levels: *ARG* by 22%, *ADC* by 31%, *ODC* by 34%, *NCPAH* by 27%, *SPDS* by 23%, *SPMS* by 35%, and *DAO* by 38%.

In contrast to the roots, the shoots showed a greater response to 100 mM Spd treatment, affecting transcript levels of polyamine metabolism-related genes more profoundly. 4 days after 100 mM Spd treatment, there was a reduction in transcript levels of *ARG* by 33%, *AIH* by 40%, *NCPAH* by 33%, *SPDS* by 29%, *SPMS* by 20%, *DAO* by 23%, and *PAO* by 46%.

The reduction in *DAO* transcript levels (by 26%) following 100 mM Spd treatment persisted for 8 days, and at this time point, a 27% decrease in *ODC* transcript levels was also observed. By 15 days post-treatment, the mRNA level of *ODC* remained down by 18%, and the transcript level of *ADC* decreased by 26%.

#### 3.2.3 The effect of spermidine treatment on H_2_O_2_ level

In the roots of plants treated with Spd, a 1.6-fold increase in H_2_O_2_ levels was observed 3 days after treatment with 100 mM Spd, and a 1.7-fold increase was seen at day 4, compared to untreated plants (**Figure 4D**). 8 days after Spd treatment, H_2_O_2_ levels rose 1.6-fold following treatment with 10 mM Spd and 1.8-fold following treatment with 100 mM Spd. Spd treatment did not induce significant changes in H_2_O_2_ levels in the shoots of treated plants compared to untreated plants.

### 3.3 The effect of spermidine treatment on infection in flax

#### 3.3.1 The effect of spermidine treatment on infection progression in flax

**Figure 5:**
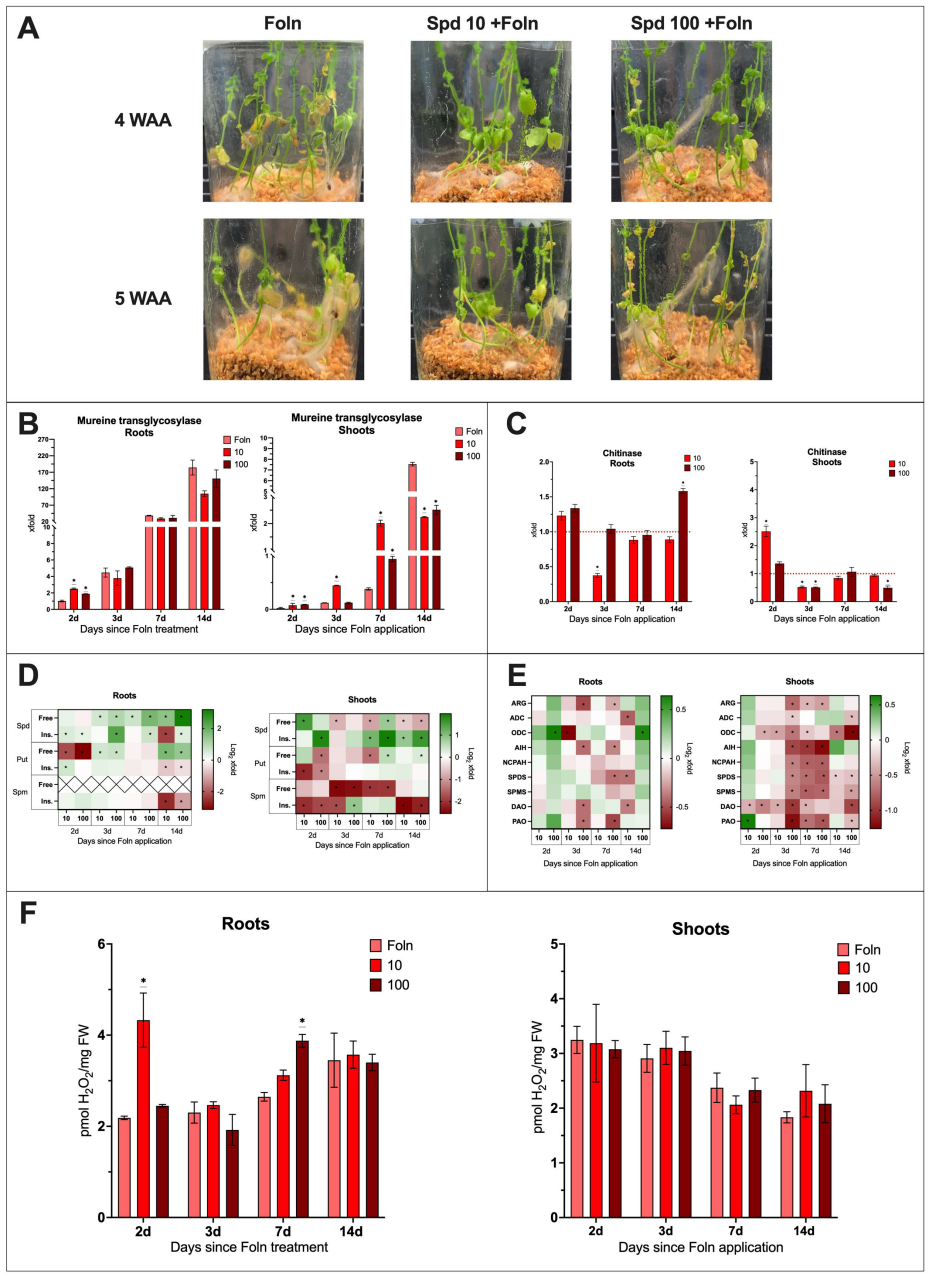
The effect of Spd treatment and *F oxysporum* infection on flax plants. **(A)** Phenotypic changes in plants treated with spermidine [10 mM and 100 mM] and infected with *F oxysporum* (WAA, weeks after application). **(B)** Relative quantity of fungal *murein transglycosylase* gene in roots and shoots of plants after Spd treatment [mM] and Foln application. The data presented were obtained from real-time PCR analysis. Flax *actin* served as the reference gene, and the relative quantity of the *murein transglycosylase* gene was normalized to untreated infected roots 2 days after Foln application. Bars represent the mean ± SD from three replicates. The significance of differences between groups was determined using two-way ANOVA followed by Tukey’s *post hoc* test (*P < 0.05 for comparison to control from the same time point as the sample). **(C)** Transcript level of *chitinase* gene in flax roots and shoots after Spd treatment [mM] and Foln application. The data were obtained from real-time RT-PCR analysis. *Actin* was used as a reference gene and the transcript levels were normalized to the untreated infected control plant (marked as red dot line). Bars represent the mean ± SD from three replicates. The significance of differences between groups was determined using two-way ANOVA followed by Tukey’s *post hoc* test (*P < 0.05 for comparison to control from the same time point as the sample). **(D)** Polyamine (SPD, spermidine; PUT, putrescine; SPM, spermine) content in flax roots and shoots after Spd treatment and Foln application. Heat map box represent the mean from three replicates. The significance of differences between groups was determined using two-way ANOVA followed by Tukey’s *post hoc* test (*P < 0.05 for comparison to control from the same time point as the sample). **(E)** Transcript level of polyamine metabolism genes in flax roots and shoots after Spd treatment and Foln application. The data were obtained from real-time RT-PCR analysis. *Actin* was used as a reference gene and the transcript levels were normalized to the untreated infected plant. Heat map box represent the mean from three measurements. The significance of the differences was determined using two-way ANOVA with Tuckey *post hoc* test (*P<0,05) (ARG, arginase; ADC, arginine decarboxylase; ODC, ornithine decarboxylase; AIH, agmatine iminohydrolase; NCPAH, N-carbamoylputrescine amidohydrolase; SPDS, spermidine synthase; SPMS, spermine synthase; DAO, diamine oxidase; PAO, polyamine oxidase). **(F)** H_2_O_2_ content in flax roots and shoots after Spd treatment [mM] and Foln application. Bars represent the mean ± SD from three replicates. The significance of differences between groups was determined using two-way ANOVA followed by Tukey’s *post hoc* test (*P < 0.05 for comparison to control from the same time point as the sample).

Phenotypic observations showed that 4 weeks after the application of Foln, plants treated with 10 mM spermidine exhibited better health compared to the infected non-treated plants. These plants showed no yellow leaves and had not yet begun to wilt (**Figure 5A**). 5 weeks after pathogen application, plants treated with 10 mM Spd were still in better visual condition compared to untreated infected plants. When plants were treated with 100 mM Spd the phenotypic changes were similar to those observed for 10 mM at 4 weeks. At 5 weeks plants treated with 100 mM were in worse condition that those treated with lower spermidine concentration but still in better health that infected untreated plants.

In the roots of plants treated with Spd and subsequently infected with Foln, 2 days after pathogen application, the level of *murine transglycosylase* gene increased 2.6-fold following treatment with 10 mM Spd and 1.9-fold after treatment with 100 mM Spd compared to untreated infected control (**Figure 5B**). At subsequent time points, the level of *murine transglycosylase* gene in the examined tissue was similar to that of the untreated infected control.

In the shoots of plants treated with 10 mM Spd and infected with Foln, there was an increase in the level of *murine transglycosylase* gene at 2, 3, and 7 days post-pathogen application, with respective increases of 4.9-fold, 3.8-fold, and 5.3-fold compared to untreated infected control plants. Similarly, in the shoots of plants treated with 100 mM Spd and infected with Foln, the *murine transglycosylase* gene level increased 4.8-fold 2 days post-infection and 2.7-fold 7 days after pathogen application.

14 days post-infection, treatment with both concentrations led to a reduction in fungal DNA levels in the infected shoots—by 70% after 10 mM Spd treatment and by 67% after 100 mM Spd treatment.

In the roots of plants treated with 10 mM Spd and infected with the pathogen, 3 days after Foln application, there was a 63% reduction in the transcript level of the *CHIT* gene compared to the untreated infected control (**Figure 5C**). 14 days after pathogen application, treatment with 100 mM Spd resulted in a 1.6-fold increase in the *CHIT* gene transcript level in infected roots.

2 days post-infection, treatment with 10 mM Spd caused a 2.5-fold increase in *CHIT* mRNA levels in infected shoots compared to the untreated infected control. However, one day later, there was a 49% decrease in the mRNA level of this gene. The same 49% reduction occurred on day 3 after pathogen application following treatment with 100 mM Spd, and a 50% decrease was also observed 14 days after pathogen application with 100 mM Spd treatment.

#### 3.3.2 The effect of spermidine treatment and infection on polyamines level

To explain the impact of spermidine treatment on the *F. oxysporum* infection of flax, the following data were described as the fold change in Spd-treated and infected plants compared to untreated and infected plants (**Figure 5D**). The absolute levels are presented in supplementary files (**Supplementary Figure S6**).

In the roots of plants treated with Spd and infected with Foln, there was an increase in the level of free spermidine compared to the untreated infected control. 3 days after Foln application, treatment with 10 mM Spd caused a 1.5-fold increase, and treatment with 100 mM Spd resulted in a 2-fold increase in free Spd levels. 7 days after Foln application, 10 mM Spd treatment led to a 1.6-fold increase, while 100 mM Spd treatment caused a 3.4-fold increase in free Spd. 14 days after Foln application, treatment with 10 mM Spd resulted in a 2.9-fold increase, and treatment with 100 mM Spd caused a 9.2-fold increase in free Spd levels.

Treatment with 100 mM Spd also led to an increase in the level of insoluble spermidine conjugates in infected roots at all examined time points (a 1.3-fold increase 2 days after Foln application, a 4.7-fold increase 3 days after Foln application, a 1.6-fold increase 7 days after Foln application, and a 1.4-fold increase 14 days after Foln application). Treatment with 10 mM Spd caused a 1.4-fold increase in the level of insoluble conjugates in infected roots 2 days after Foln application; however, 14 days after Foln application, there was an 80% decrease in this fraction of Spd compared to untreated infected plants.

In the shoots of plants treated with 10 mM Spd and infected with Foln, there was an initial 3-fold increase in the level of free spermidine. However, as the infection progressed, treatment with 10 mM Spd caused a decrease in the level of this spermidine fraction in the infected shoots (41% decrease 3 days after Foln application, a 46% decrease 7 days after Foln application, and a 30% decrease 14 days after Foln application). 7 days after Foln application, treatment with 100 mM Spd resulted in a 1.8-fold increase in free spermidine levels. However, 14 days after Foln application, this concentration led to a 45% decrease in free spermidine levels in infected shoots compared to the untreated infected control.

Treatment with Spd caused an increase in the level of insoluble spermidine conjugates in the infected shoots. 2 days after Foln application, treatment with 100 mM Spd caused a 3.6-fold increase in the level of insoluble spermidine conjugates. 7 days after Foln application, treatment with 10 mM Spd resulted in a 2.2-fold increase, while treatment with 100 mM Spd caused a 4-fold increase in the level of insoluble spermidine conjugates. 14 days after Foln application, treatment with 10 mM Spd caused a 1.8-fold increase, and treatment with 100 mM Spd resulted in a 3-fold increase in the level of insoluble spermidine conjugates in infected shoots.

Treatment with Spd initially significantly reduced the level of free Put in infected roots. Treatment with 10 mM Spd resulted in a 78% decrease, and 100 mM Spd caused an 89% decrease in free Put compared to untreated infected roots. However, 3 days after Foln application, treatment with 10 mM Spd led to a 1.5-fold increase in free Put levels, while treatment with 100 mM Spd resulted in a 1.6-fold increase. 14 days after pathogen application, the level of free Put was elevated, with a 3.4-fold increase following treatment with 10 mM Spd and a 2.6-fold increase with 100 mM Spd.

Treatment with 10 mM Spd caused a 1.5-fold increase in the level of insoluble Put conjugates 2 days after pathogen application in roots compared to untreated infected plants. However, 14 days after pathogen application, there was a decrease in this fraction of Put in infected roots, with a 37% decrease following 10 mM Spd treatment and a 25% decrease after 100 mM Spd treatment.

7 days after Foln application, treatment with 10 mM Spd caused a 61% decrease in the level of free Put in infected shoots compared to untreated infected plants. Treatment with 100 mM Spd also reduced the level of free Put by 62% 2 days after Foln application. However, 7 and 14 days after Foln application, an increase in free Put levels was observed after treatment with 100 mM Spd—1.5-fold and 1.2-fold, respectively.

In infected shoots, the level of insoluble Put conjugates decreased two days after Foln application by 77% following treatment with 10 mM Spd and by 54% after treatment with 100 mM Spd.

Following Spd treatment, a sharp decrease in the level of insoluble Spm conjugates was observed in infected roots 14 days after Foln application compared to untreated infected plants. Treatment with 10 mM Spd caused an 83% decrease, while 100 mM Spd caused a 56% decrease. In infected shoots, treatment led to a reduction in free Spm levels. 3 days after Foln application, treatment with 10 mM Spd caused an 82% reduction, and 100 mM Spd caused an 81% reduction in free Spm levels in infected shoots compared to the untreated infected control. 7 days after Foln application, a 74% reduction was caused by 10 mM Spd treatment, while 100 mM Spd caused a 75% reduction in free Spm. Treatment with 10 mM Spd resulted in a decrease in the level of insoluble Spm conjugates 2 days (77% decrease), three days (71% decrease), and 14 days (82% decrease) after Foln application. Treatment with 100 mM Spd led to a decrease in insoluble Spm conjugates 2 and 14 days after Foln application (72% and 79% decreases, respectively). However, 3 days after Foln application, treatment with 100 mM Spd caused a 1.8-fold increase in this Spm fraction in infected shoots.

#### 3.3.3 The effect combination of spermidine treatment and infection on the transcript level of polyamine metabolism genes

To explain the impact of spermidine treatment on the *F. oxysporum* infection of flax, the following data were described as the fold change in Spd-treated and infected plants compared to untreated and infected plants (**Figure 5E**; **Supplementary Figures S7**, **S8**).

Treatment with Spd did not induce many significant changes in the roots of infected plants. The 100 mM Spd treatment increased the transcript level of only the *ODC* gene in the roots at 2 days (1.6-fold increase) and 14 days (1.5-fold increase) after Foln application. However, 3 days post-Foln application, treatment with 10 mM Spd reduced the *ODC* transcript level by 40%. 14 days after Foln application, treatment with 10 mM Spd in infected roots led to a decrease in the mRNA levels of *ADC* (by 28%), *SPDS* (by 24%), and *DAO* (by 23%). The 100 mM Spd treatment also resulted in reduced transcript levels of certain genes in infected roots at both 3 and 7 days post-Foln application. At 3 days after Foln, the transcript levels of *ARG*, *AIH*, *DAO*, and *PAO* were reduced by 31%, 25%, 25%, and 25%, respectively. By 7 days post-Foln, there was a decrease in the levels of *ARG* (by 16%), *AIH* (by 22%), *SPDS* (by 25%), and *PAO* (by 31%).

Treatment with 10 mM Spd led to a 1.5-fold increase in the transcript level of the *PAO* gene in infected shoots 2 days after Foln application. At the same time point, there was also a 24% reduction in the transcript level of the *DAO* gene. Few changes were also observed 3 days after pathogen application following treatment with 10 mM Spd, including a 28% decrease in the *ODC* gene transcript and a 26% decrease in the *DAO* gene transcript.

In the infected shoots, 7 days after Foln application, treatment with 10 mM Spd lowered the transcript levels of most genes associated with polyamine metabolism: *ARG* by 23%, *ODC* by 18%, *AIH* by 50%, *NCPAH* by 42%, *SPDS* by 40%, *SPMS* by 43%, *DAO* by 24%, and *PAO* by 39%. Even 14 days after pathogen application, the transcript levels of *ODC* (reduction by 38%) and *SPDS* (reduction by 15%) remained lowered following treatment with 10 mM Spd.

2 days after Foln application, treatment with 100 mM Spd resulted in decreased transcript levels of the *ODC* (25% reduction) and *DAO* (35% reduction) genes in infected shoots. By the next day, a reduction in the transcript levels of all studied genes was observed: *ARG* by 41%, *ADC* by 17%, *ODC* by 38%, *AIH* by 47%, *NCPAH* by 36%, *SPDS* by 35%, *SPMS* by 36%, *DAO* by 50%, and *PAO* by 37%.

7 and 14 days after Foln application following treatment with 100 mM Spd, reduced transcript levels of several polyamine metabolism-related genes were still observed in infected shoots. 7 days post-application, the transcript levels of *ARG* (28% reduction), *AIH* (57% reduction), *NCPAH* (43% reduction), *SPDS* (44% reduction), *SPMS* (41% reduction), and *PAO* (44% reduction) were lowered. 14 days after pathogen application, the transcript levels of *ADC* (22% reduction), *ODC* (59% reduction), *SPDS* (20% reduction), *SPMS* (28% reduction), *DAO* (47% reduction), and *PAO* (21% reduction) were still reduced in infected shoots.

#### 3.3.4 The effect of spermidine treatment and infection on H_2_O_2_ level

2 days after Foln application, treatment with 10 mM Spd led to a 2-fold increase in H_2_O_2_ levels in the roots compared to untreated, infected roots. A 1.5-fold increase in H_2_O_2_ levels was also observed 7 days after Foln application as a result of treatment with 100 mM Spd. In the shoots of treated and infected plants, no significant differences were observed compared to the shoots of untreated plants infected with Foln (**Figure 5F**).

## 4 Discussion

Our study aimed to investigate the role of spermidine (Spd) in modulating polyamine metabolism and stress response in flax (*Linum usitatissimum*) under *Fusarium oxysporum* infection. Our aim was to elucidate how exogenous spermidine may affect plant immunity and contribute to developing sustainable, polyamine-based strategies for protecting flax against fusarium wilt. Our key findings indicate that Spd treatment significantly influenced the polyamine profile in flax plants, but the levels varied depending on the concentration used, the time of treatment, and the investigated tissue, and this effect can be attributed to both the uptake of the exogenous metabolite and endogenous metabolism. Spd application changed the expression of genes related to polyamine metabolism, which are crucial in maintaining polyamine homeostasis. Concurrently, Spd treatment mitigated the progression of *Fusarium* infection, an effect that may be attributed to free polyamines in roots and cell wall bound polyamines in shoots.

Polyamines, as multifunctional compounds, have been recognized for their potential in enhancing plant resilience against pathogens. However, their effects vary across pathosystems, often depending on the treatment concentration and plant species. They interact with cellular components to stabilize structures under stress and are implicated in defence signalling through their involvement in oxidative stress regulation. Understanding these interactions is crucial, as polyamines may both enhance and suppress plant defences.

Our findings align with previous research demonstrating the role of spermidine in enhancing stress tolerance in plants, including wheat, maize, and *Arabidopsis*. Additionally, our previous research conducted on another flax cultivar (Jan) indicated similar, but not identical, changes (Augustyniak et al., 2024). The Jan cultivar exhibited much more pronounced changes in free Spd levels in the roots following treatment with both 10 mM and 100 mM Spd, while in the Nike cultivar, only treatment with 100 mM Spd caused sustained increases in free spermidine levels over a prolonged period, while treatment with 10 mM Spd only led to a transient rise. Our data not only demonstrate that flax absorbs spermidine from the medium, but also reveal interesting differences among various cultivars within the same species. The literature indicates that other plant species also take up exogenous polyamines both via roots (as seen in hydroponic cultivation of maize and wheat supplemented with Put, Spd, or Spm (Szalai et al., 2017)) and via leaves, as demonstrated in *Arabidopsis*, which was sprayed with 500 μM Spd or Spm (van Rensburg et al., 2021).

In our experiments, following the 100 mM Spd treatment, free spermidine levels remained elevated over a prolonged period, while transcript levels of many polyamine-related genes were downregulated. UPLC data showed that free putrescine increased on day 8, followed by a decrease, particularly in roots. Specifically, putrescine synthesis via arginine was downregulated, while the branch involving ornithine was strongly activated. However, no changes were observed in the transcript levels of *spermine* and *spermidine synthesis* genes, while the genes responsible for PA degradation showed reduced expression. Hence, it can be concluded that increased spermidine levels are mainly related to its uptake from the medium and limited degradation.

In shoots, both concentrations induced a sharp initial increase, followed by a rapid decrease in free spermidine, implying a different regulatory mechanism compared to roots. The gene expression data similarly showed an early decrease in the transcript levels of biosynthetic genes such as *SPDS* and *SPMS*. This might indicate that shoots, unlike roots, quickly downregulate endogenous polyamine biosynthesis in response to excess spermidine.

Studies using radioactively labelled polyamines demonstrate the diverse transport mechanisms of these compounds between plant organs. For example, ^15^N-putrescine incubated with soybean seeds was subsequently found in the soybean cotyledons. During development, a decrease in the amount of ^15^N-putrescine in the cotyledons was observed, alongside an increase in the roots and shoots (Ohe et al., 2005). Similarly, in the Jan cultivar, it was observed that 3–4 days after Spd treatment, the level of free spermidine in the roots remained elevated, but not to the same extent as observed 2 days after treatment, and a rise in free spermidine levels was noted in the shoots on days 3–4 after treatment (Augustyniak et al., 2024).

The levels of conjugated polyamines, particularly spermidine and putrescine, fluctuated significantly after Spd treatment. The accumulation of insoluble spermidine conjugates in roots treated with 100 mM Spd indicates long-term conjugation processes, particularly noticeable 15 days after treatment. The increase in conjugated putrescine at later stages (day 15) suggests a similar mechanism to spermidine conjugation. In previous studies on the Jan cultivar, Spd treatment also led to a rapid increase (2 days post-treatment) in both free and insoluble conjugates of putrescine. However, no changes were observed in the transcript levels of genes involved in the biosynthesis of this polyamine (Augustyniak et al., 2024). Interestingly, in Nike flax shoots, conjugated spermidine levels eventually declined at both Spd concentrations, highlighting different tissue responses to spermidine over time.

This conjugation may serve as a regulatory mechanism to buffer excess free polyamines, but further investigation is needed. The sustained increase in conjugated spermidine aligns with decreased mRNA levels of *PAO* and *DAO*, suggesting that conjugation pathways rather than degradation are employed when the plant is trying to manage excess polyamines. By downregulating these genes, plants might be intentionally slowing the breakdown of polyamines and its connected production of H_2_O_2_. The downregulation of *SPDS* and *SPMS* genes also suggests that the plant is curbing the biosynthesis of new polyamines, likely to prevent further accumulation, while conjugation remains active.

Studies on maize have shown that excess putrescine triggers a response similar to a wound response, associated with membrane depolarization and resulting in membrane damage. This reaction was linked to the activity of DAO, leading to the breakdown of excess putrescine, which subsequently caused oxidative stress and membrane damage (DiTomaso et al., 1989). The downregulation of *NCPAH*, involved in putrescine metabolism, further supports the idea of limiting active polyamine production and shifting the focus towards conjugation.

In *Agrostis stolonifera*, endogenous Spm levels increased following treatment with Spd (Li et al., 2015). In rice panicles treated with 1 mM Spd for 5 days, free Put and Spd levels decreased 5 days after the treatment ended, while free Spm levels increased. This shift was accompanied by elevated transcript levels of the *OsODC1* and *OsSPDS3* genes, while no changes were detected in the expression of *OsADC1* and *OsSPDS1* (Zhou et al., 2020). This highlights that while all plant species respond to treatments, the end results can be vastly different.

Treatment with 100 mM Spd resulted in an increase in H_2_O_2_ levels in the roots at 3, 4, and 8 days post-treatment compared to untreated control plants. No significant changes were observed in the shoots. Although H_2_O_2_ is generated during polyamine catabolism, as previously mentioned, the transcript levels of the *DAO* and *PAO* genes were reduced in both examined tissues following treatment. Clearly, 100 mM Spd treatment caused oxidative stress, and 10 mM Spd did not. Lower concentrations of Spd (100 and 500 µM) applied in studies on *Arabidopsis* did not lead to changes in H_2_O_2_ levels (van Rensburg et al., 2021), similar to the effects observed with 10 mM Spd in this study. Excess H_2_O_2_ can be damaging for plants, but it also serves as a signalling molecule. Both concentrations were used in our studies for comparison.

While changes in both polyamines and genes fluctuated in flax plants, this treatment influenced not only the progression of infection but also how the polyamine pathway responded to *Fusarium* infection, as changes were more pronounced when infected plants were treated with Spd.

The progression of infection was confirmed by phenotypic observations, as several weeks post-application of *F. oxysporum*, plants exhibited typical fusarium wilt symptoms, such as yellowing and wilting of the leaves. The phenotypic changes were not visible at earlier time points; however, they correlate well with the plant’s response at the molecular level at earlier stages. At all times, fungal presence was verified by measuring the level of fungal *murein transglycosylase* gene, which showed a gradual increase in both roots and shoots over time. The mRNA levels of the *chitinase* gene were significantly higher in the roots of infected plants compared to uninfected controls, indicating a strong activation of defence mechanisms in response *to F. oxysporum.* In the shoots, *chitinase* mRNA levels initially decreased, followed by an increase, reflecting a delayed but notable defensive response in the aerial parts of the plant, which corresponds to the later stage at which the fungus penetrates this tissue. Previous studies on the Jan cultivar demonstrated a similar pattern of *chitinase* gene behaviour in both the roots and shoots of infected plants (Augustyniak et al., 2024).

After Spd treatment on day 14 post-infection, the fungal DNA levels had dropped significantly (by 70% in shoots treated with 10 mM Spd and 67% with 100 mM Spd). This suggests that Spd treatment helped suppress fungal growth, particularly at later stages, by possibly enhancing the plant’s ability to resist infection. This can be due to the direct effect of polyamines on the fungus, as we demonstrated earlier that they can inhibit fungal growth *in vitro* (Wojtasik et al., 2015), strengthening the plant cell wall by accumulation of cell-bound polyamines and influencing the plant defence response via expression of PR genes. A study on the Jan cultivar showed that treatment with 100 mM Spd resulted in a quick increase in the mRNA level of *chitinase* in infected roots (Augustyniak et al., 2024). Similar results were obtained when examining *Botrytis cinerea* infection in *Arabidopsis thaliana*. It was shown that treatment with 10 mM Spm induced systemic-acquired resistance gene responses, including *chitinase* (Seifi et al., 2019). However, in this study, in Spd-treated plants, the response of *chitinase* was more complex. In roots treated with 10 mM Spd, *chitinase* transcript levels were initially suppressed (63% decrease by day 3) but showed an increase with 100 mM Spd treatment by day 14 (1.6-fold). In shoots, *chitinase* mRNA initially increased (2.5-fold by day 2) but was then sharply reduced by nearly 50% by day 3 and day 14. Based on our previous and current studies, we can conclude that the level of *chitinase* transcript depends on the amount of fungus in the plant. However, in the roots of plants treated with 100 mM Spd and infected with Foln, there was a comparable amount of fungus, and yet an increase in *chitinase* transcript was observed. This is most likely the result of oxidative stress observed in these plants at the earlier time points, as oxidative stress itself can increase *chitinase* mRNA. Zheng et al (2004). demonstrated that PR protein activity in *Capsicum annuum* roots was modulated by infection with *Phytophthora capsici* and correlated with oxidative enzyme activity and H_2_O_2_ levels. Similarly, Lin et al (2005). observed that hydrogen peroxide acts as a signalling molecule mediating *chitinase* gene expression in rice, highlighting its central role in plant defence responses triggered by stress or elicitors (Zheng et al., 2004; Lin et al., 2005).

When flax plants are infected with *Fusarium*, substantial changes are observed, mainly in the conjugated forms of polyamines, but also in free putrescine and free spermine in the shoots at early time points. In plants treated with spermidine and subsequently infected, there was an increase in the levels of both free and insoluble conjugates of spermidine in the roots, as well as in insoluble conjugates of spermidine in the shoots. In the case of the roots, both concentrations appeared to yield similar effects; however, for the shoots, the higher concentration resulted in a more rapid increase in the levels of insoluble conjugates.

Following an initial decline, the level of free putrescine in the roots also increased. 14 days post-application of Foln, treatment with both concentrations resulted in a noticeable decrease in the levels of insoluble conjugates of putrescine and spermine. Significant reductions in both spermine fractions were also observed in the shoots. This observation is particularly interesting in light of the changes in the levels of this polyamine in untreated plants infected with Foln, suggesting that spermidine may be more effective in combating the infection than spermine. Consequently, the increase in spermidine levels led to a reduction in spermine levels. Moreover, the decrease in the expression of various polyamine biosynthesis and catabolism genes following spermidine treatment indicates that the exogenously supplied spermidine is sufficient for effective defence against the pathogen, and further synthesis is unnecessary. Without spermidine treatment, the Foln infection led to an increase in mRNA levels of several polyamine metabolism genes in flax, particularly in the roots. The efficacy of this defence is further corroborated by the improved phenotypic condition of the plants and the observed reduction in fungal DNA levels in treated plants compared to the infected control.

Our findings indicate that treatment with 10 mM Spd significantly improves plant phenotypic health compared to treatment with 100 mM Spd. Plants treated with 10 mM Spd exhibited reduced fungal murein levels, particularly in the shoots, and maintained chitinase activity at levels similar to or lower than control plants. This suggests that additional defence activation may not be necessary, as the progression of the disease appears to be halted. Notably, an oxidative burst occurred two days after pathogen exposure, followed by a restoration of redox balance, pointing to a well-regulated defence response in the treated plants. These results suggest that moderate Spd treatment could offer a viable strategy for enhancing flax resistance to fusarium wilt, with minimal impact on plant physiology, and support further exploration of polyamine-based approaches in sustainable crop protection.

While our study provides valuable insights into the role of spermidine in flax stress tolerance, several limitations should be acknowledged. First, the experiments were conducted under controlled conditions, which may not fully reflect field conditions where multiple environmental stressors interact. Second, our analysis primarily focused on polyamine metabolism and antioxidant responses, leaving other defence mechanisms, such as hormonal signalling pathways, underexplored. Future studies integrating transcriptomic and proteomic approaches could offer a more comprehensive understanding of Spd-mediated defence responses. Lastly, the long-term effects of Spd application on flax growth and yield remain unexplored and warrant further investigation.

The findings of this study have significant implications for improving flax cultivation under biotic stress conditions. The ability of spermidine to enhance resistance to *Fusarium oxysporum* highlights its potential as a biostimulant in crop protection strategies. By modulating polyamine metabolism and antioxidant responses, Spd treatment could be integrated into sustainable agricultural practices to reduce reliance on chemical fungicides. Moreover, the upregulation of key polyamine biosynthetic genes opens avenues for genetic engineering approaches aimed at developing *Fusarium*-resistant flax varieties. Future research should focus on field trials and the formulation of optimal Spd treatment strategies to maximize its protective benefits in commercial flax production.

## 5 Conclusions

Our findings indicate that treatment with 10 mM Spd notably improves plant phenotypic health compared to 100 mM Spd. Plants treated with 10 mM Spd exhibited reduced fungal *murein transglycosylase* levels, particularly in shoots, and maintained chitinase activity similar to or lower than control plants, suggesting that additional defence activation may be unnecessary. Notably, an oxidative burst occurred two days after pathogen exposure, followed by redox balance restoration, pointing to a measured defence response in the treated plants. These results suggest that moderate Spd treatment could offer a viable strategy for enhancing flax resistance to fusarium wilt with minimal impact on plant physiology, supporting further exploration of polyamine-based approaches in sustainable crop protection.

## Data Availability

The original contributions presented in the study are included in the article/**Supplementary Material**. Further inquiries can be directed to the corresponding author/s.
