# Supplementary material for: Spermidine treatment limits the development of the fungus in flax shoots by suppressing polyamine metabolism and balanced defence reactions, thus increasing flax resistance to fusariosis
Source: Front Plant Sci. 2025 Mar 25;16:1561203. doi: 10.3389/fpls.2025.1561203 (PMC11975865; doi:10.3389/fpls.2025.1561203)
Supplement: Supplementary file 1 [file DataSheet1.docx]

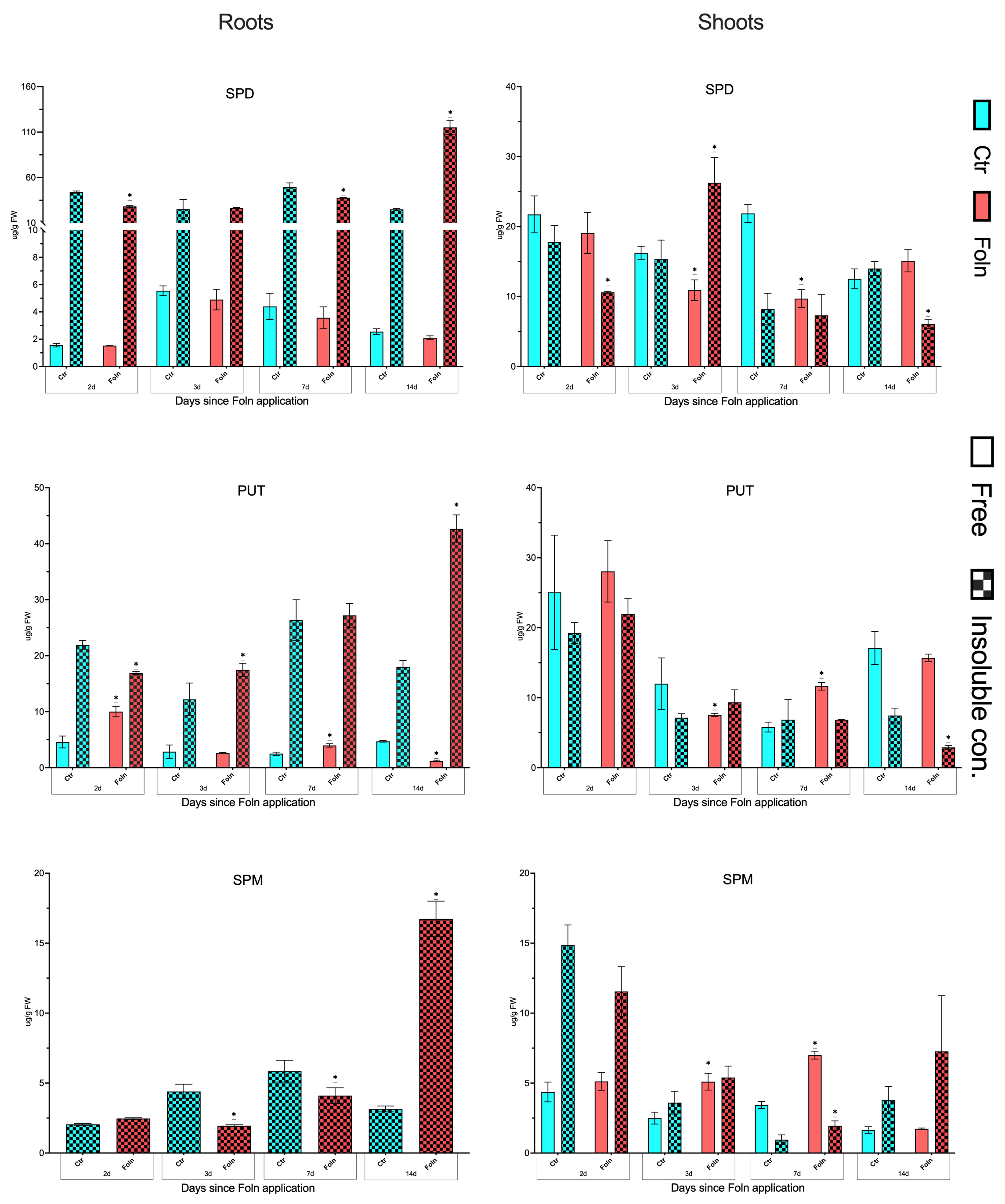


**Figure S1** Polyamine (SPD spermidine; PUT putrescine; SPM spermine) content in flax roots and shoots after *F. oxysporum* application. Bars represent the mean ± SD from three replicates. The significance of differences between groups was determined using two-way ANOVA followed by Tukey's post hoc test (*P < 0.05 for comparison to control from the same time point as the sample).


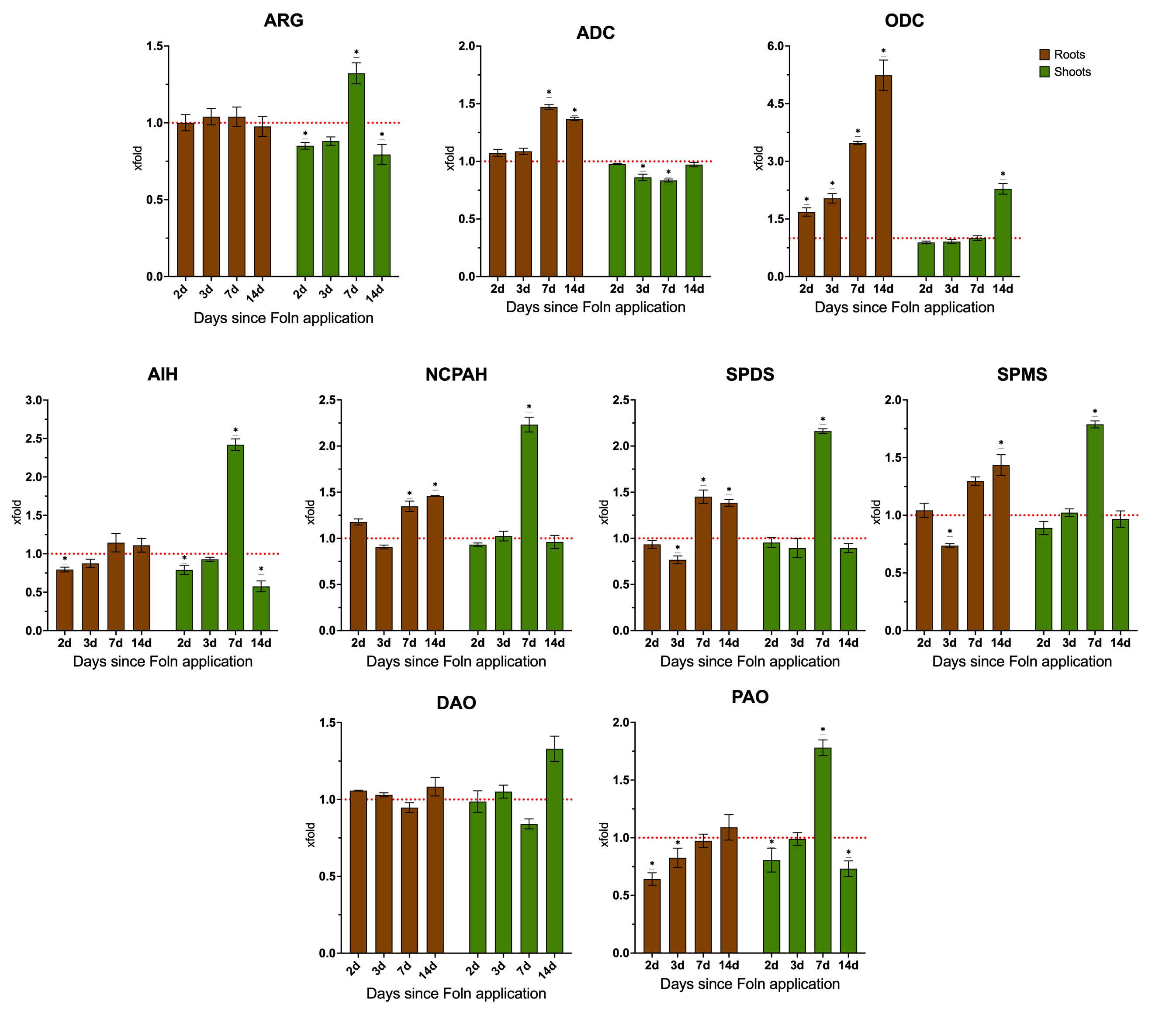


**Figure S2** Transcript level of polyamine metabolism genes in flax roots and shoots after Foln application. The data were obtained from real-time RT-PCR analysis. *Actin* was used as a reference gene and the transcript levels were normalized to the untreated control plant. Bars represent the mean ± SD from three replicates. The significance of the differences was determined using two-way ANOVA with Tuckey post hoc test (*P<0,05) (*ARG arginase; ADC arginine decarboxylase; ODC ornithine decarboxylase; AIH agmatine iminohydrolase; NCPAH N-carbamoylputrescine amidohydrolase; SPDS spermidine synthase; SPMS spermine synthase; DAO diamine oxidase; PAO polyamine oxidase*).


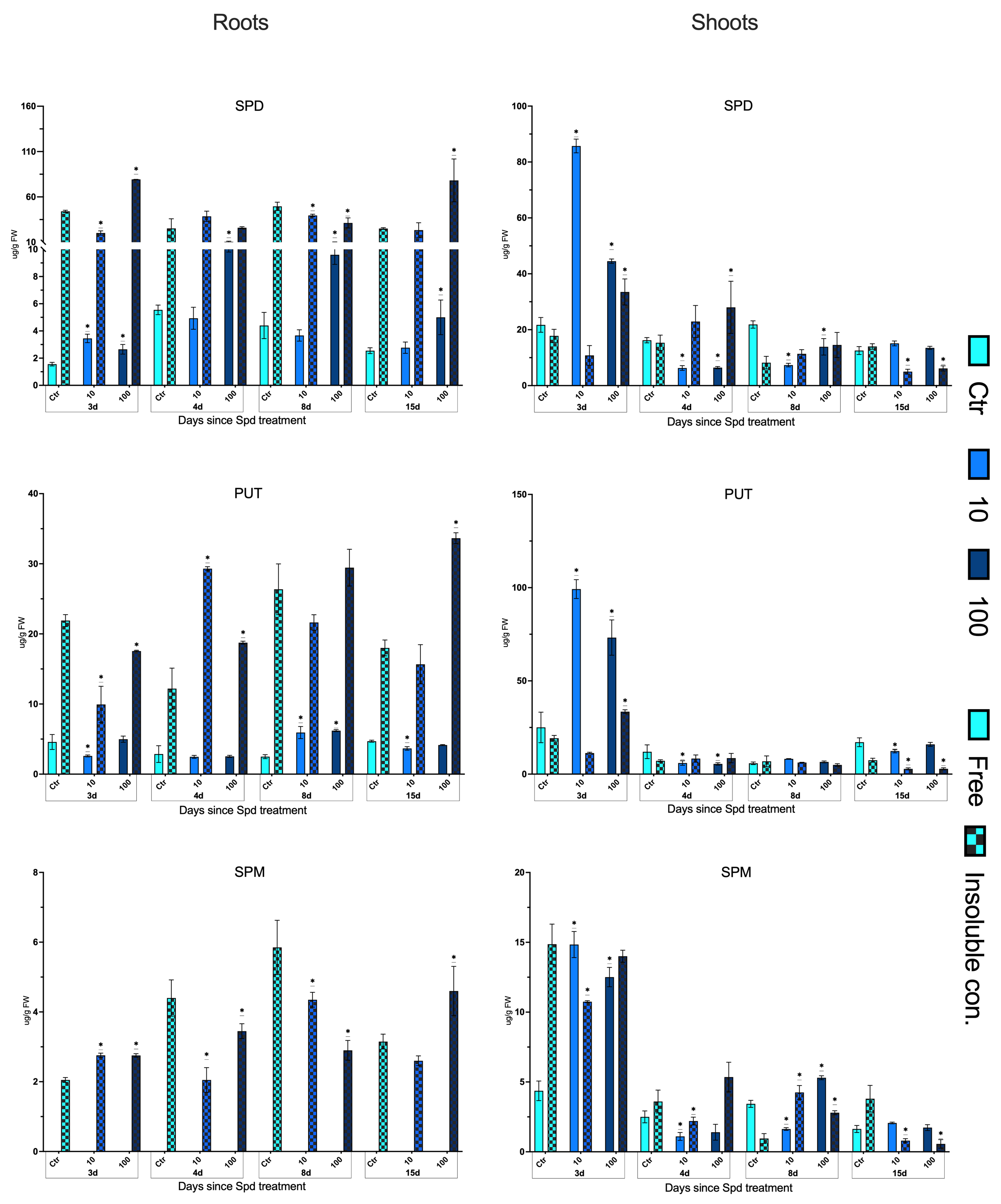


**Figure S3** Polyamine (SPD spermidine; PUT putrescine; SPM spermine) content in flax roots and shoots after spermidine treatment [10 and 100 mM]. Bars represent the mean ± SD from three replicates. The significance of differences between groups was determined using ANOVA followed by Tukey's post hoc test (*P < 0.05 for comparison to control from the same time point as the sample).


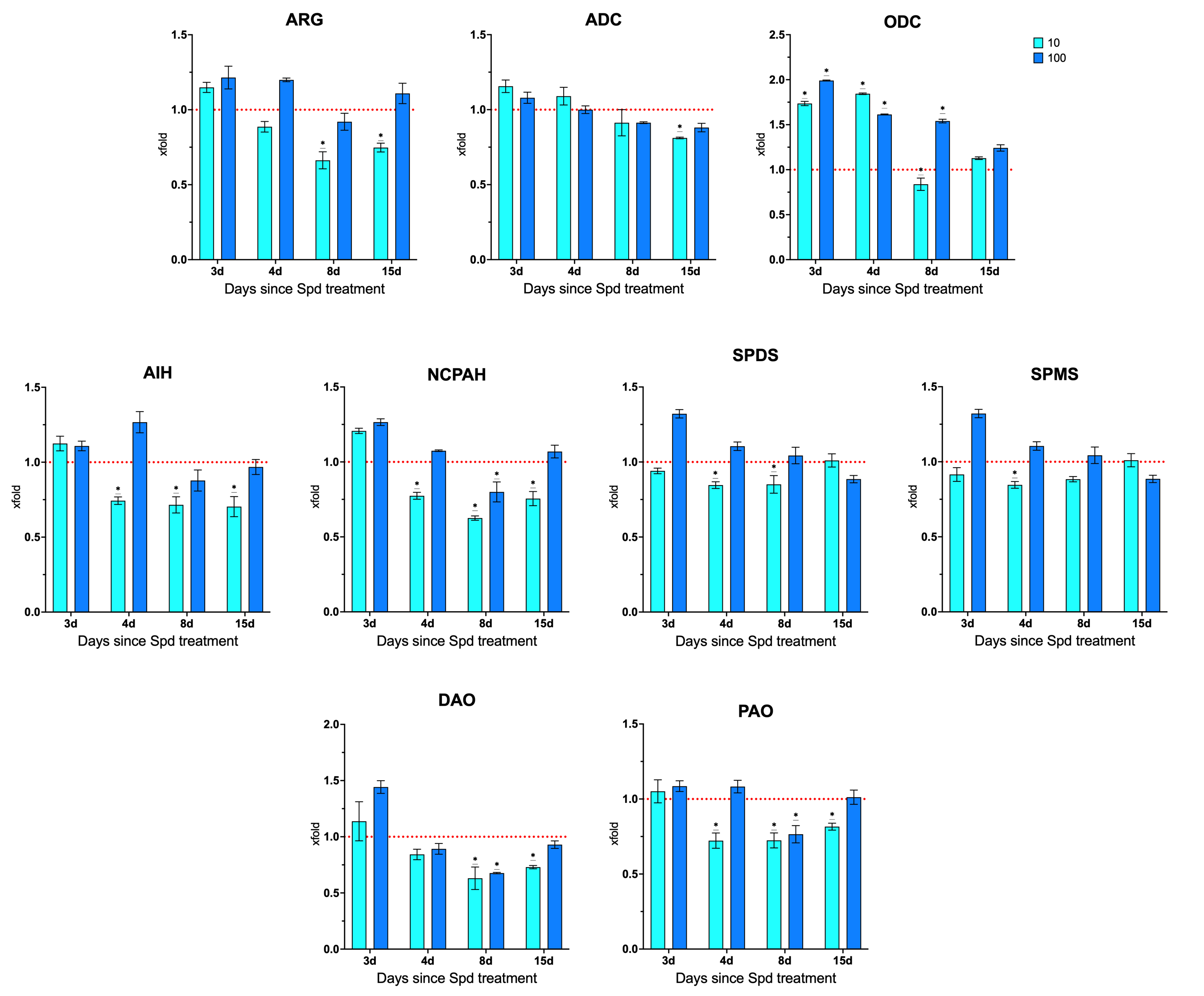


**Figure S4** Transcript level of polyamine metabolism genes in flax roots after Spd treatment [10 and 100 mM]. The data were obtained from real-time RT-PCR analysis. *Actin* was used as a reference gene and the transcript levels were normalized to the untreated control plant. Bars represent the mean ± SD from three replicates. The significance of the differences was determined using two-way ANOVA with Tuckey post hoc test (*P<0,05) (*ARG arginase; ADC arginine decarboxylase; ODC ornithine decarboxylase; AIH agmatine iminohydrolase; NCPAH N-carbamoylputrescine amidohydrolase; SPDS spermidine synthase; SPMS spermine synthase; DAO diamine oxidase; PAO polyamine oxidase*).


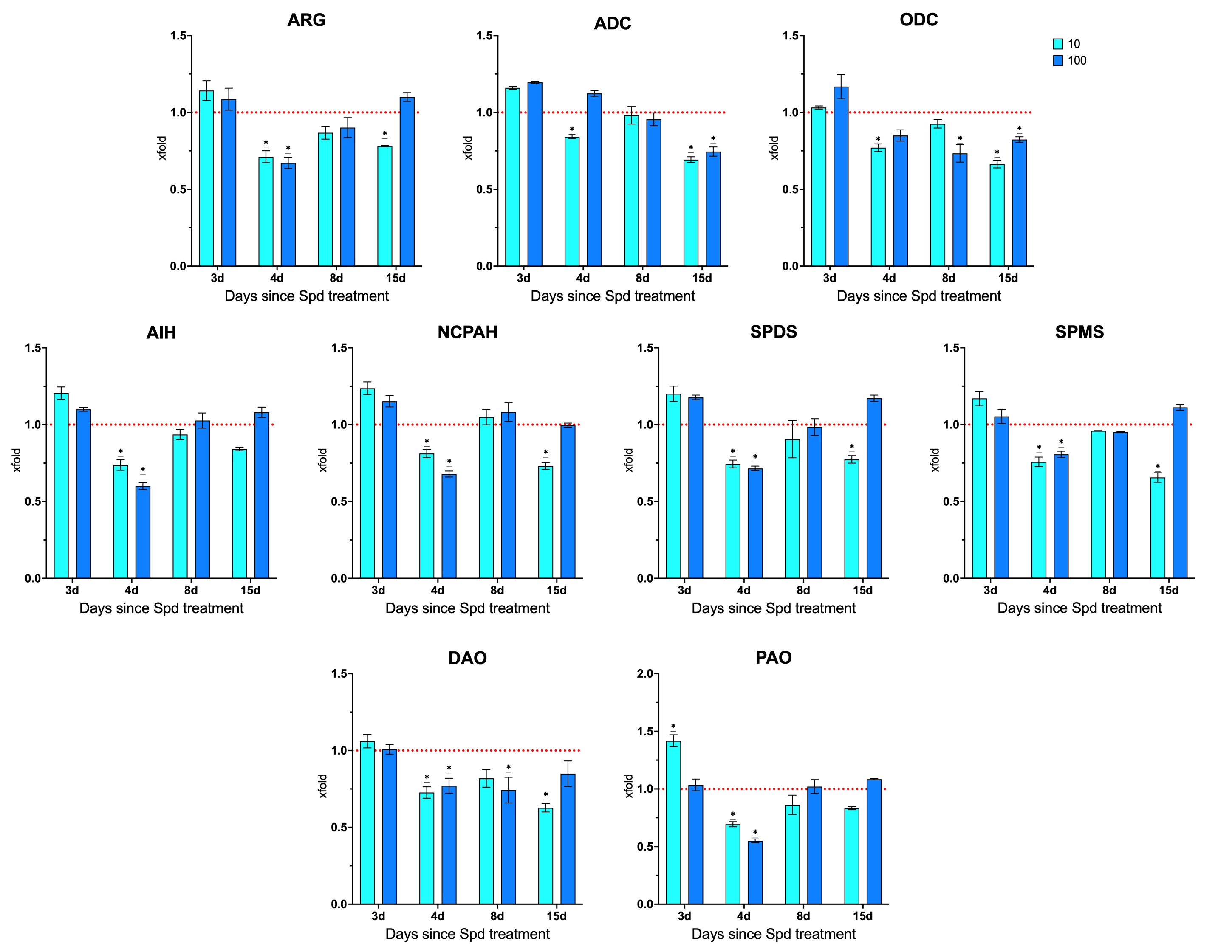


**Figure S5** Transcript level of polyamine metabolism genes in flax shoots after Spd treatment [10 and 100 mM]. The data were obtained from real-time RT-PCR analysis. *Actin* was used as a reference gene and the transcript levels were normalized to the untreated control plant. Bars represent the mean ± SD from three replicates. The significance of the differences was determined using two-way ANOVA with Tuckey post hoc test (*P<0,05) (*ARG arginase; ADC arginine decarboxylase; ODC ornithine decarboxylase; AIH agmatine iminohydrolase; NCPAH N-carbamoylputrescine amidohydrolase; SPDS spermidine synthase; SPMS spermine synthase; DAO diamine oxidase; PAO polyamine oxidase*).


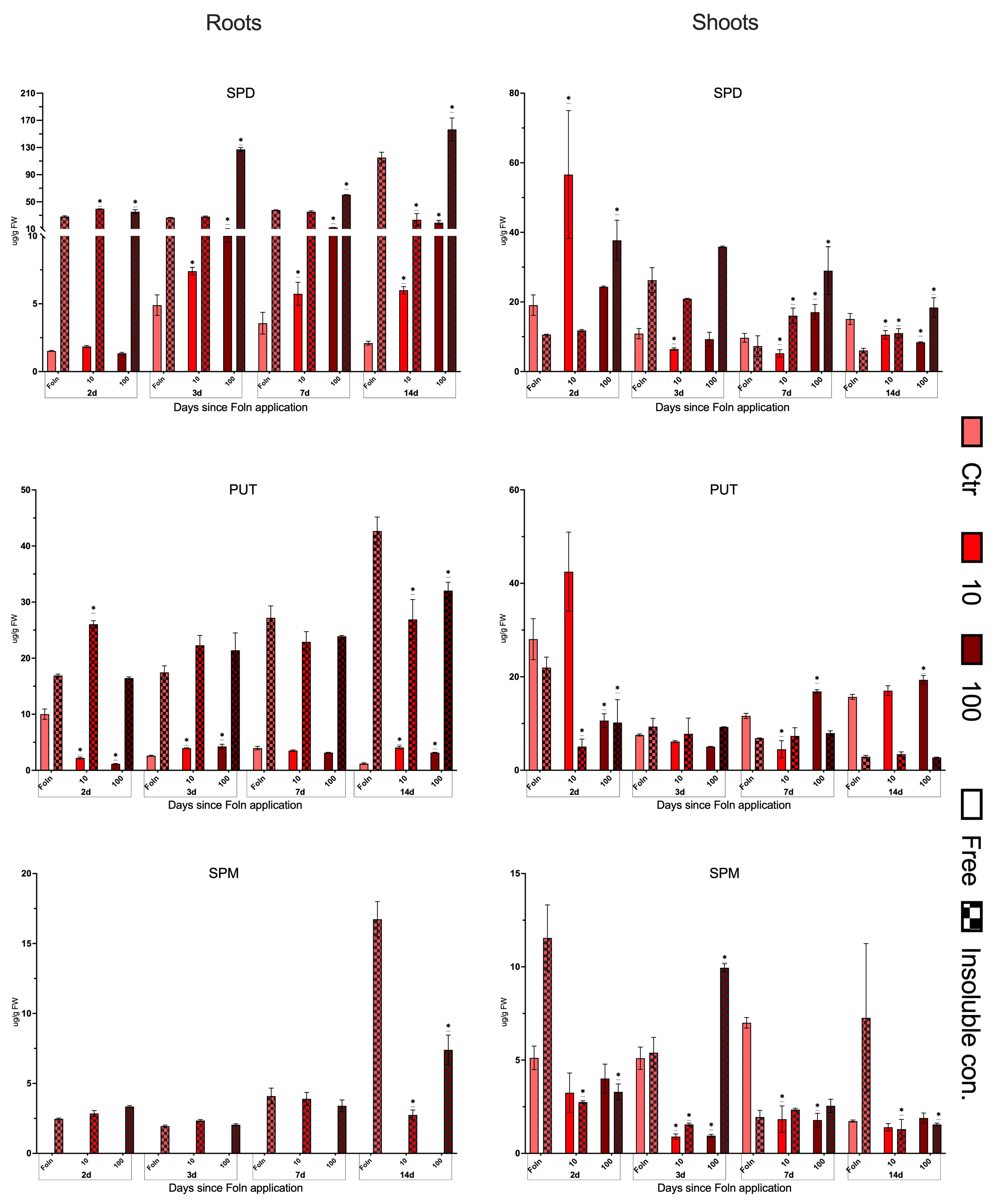


**Figure S6** Polyamine (SPD spermidine; PUT putrescine; SPM spermine) content in flax roots and shoots after spermidine treatment [10 and 100 mM] and Foln application. Bars represent the mean ± SD from three replicates. The significance of differences between groups was determined using two-way ANOVA followed by Tukey's post hoc test (*P < 0.05 for comparison to control from the same time point as the sample).


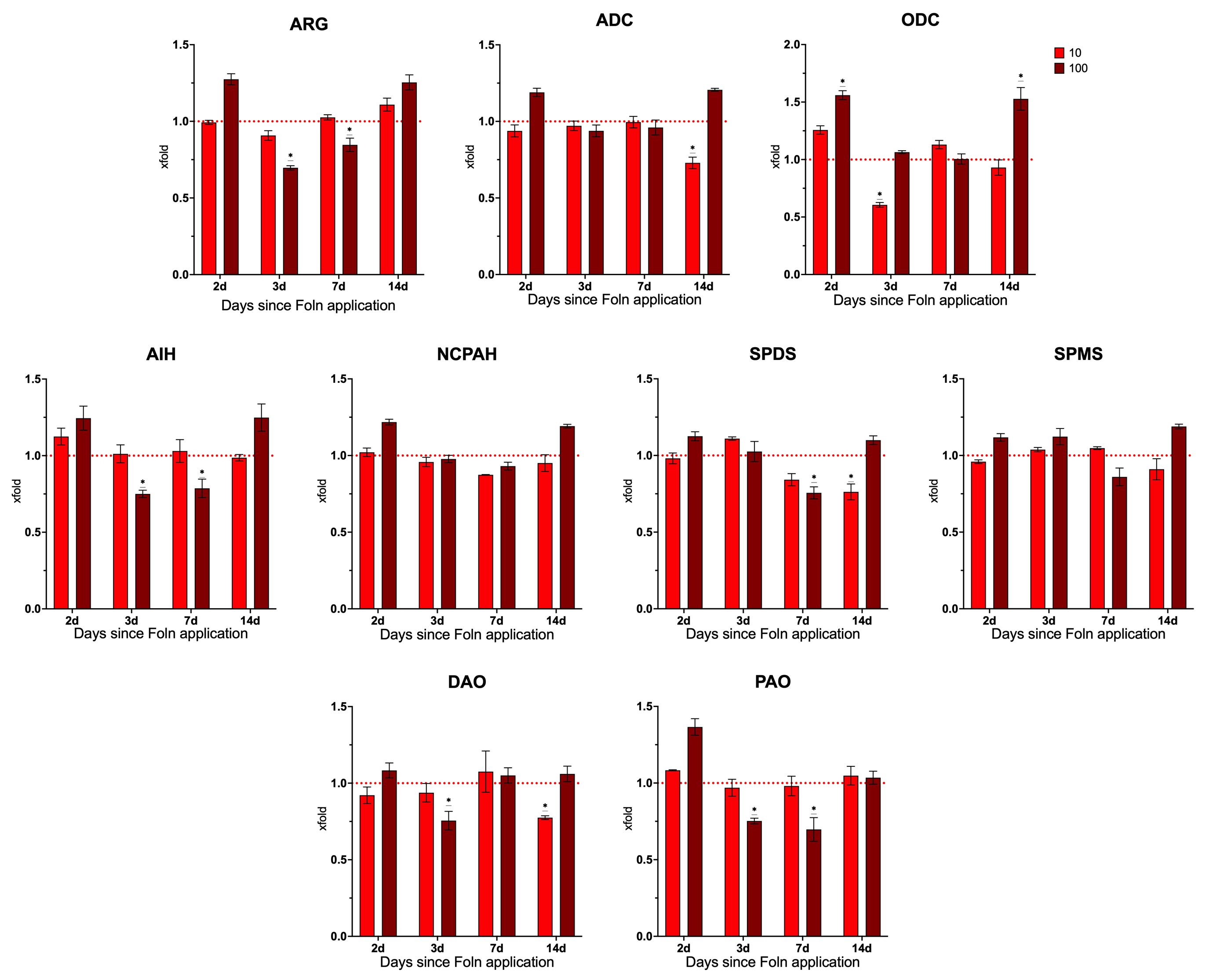


**Figure S7** Transcript level of polyamine metabolism genes in flax roots after Spd treatment [10 and 100 mM] and Foln application. The data were obtained from real-time RT-PCR analysis. *Actin* was used as a reference gene and the transcript levels were normalized to the untreated infected plant. Bars represent the mean ± SD from three replicates. The significance of the differences was determined using two-way ANOVA with Tuckey post hoc test (*P<0,05) (*ARG arginase; ADC arginine decarboxylase; ODC ornithine decarboxylase; AIH agmatine iminohydrolase; NCPAH N-carbamoylputrescine amidohydrolase; SPDS spermidine synthase; SPMS spermine synthase; DAO diamine oxidase; PAO polyamine oxidase*).


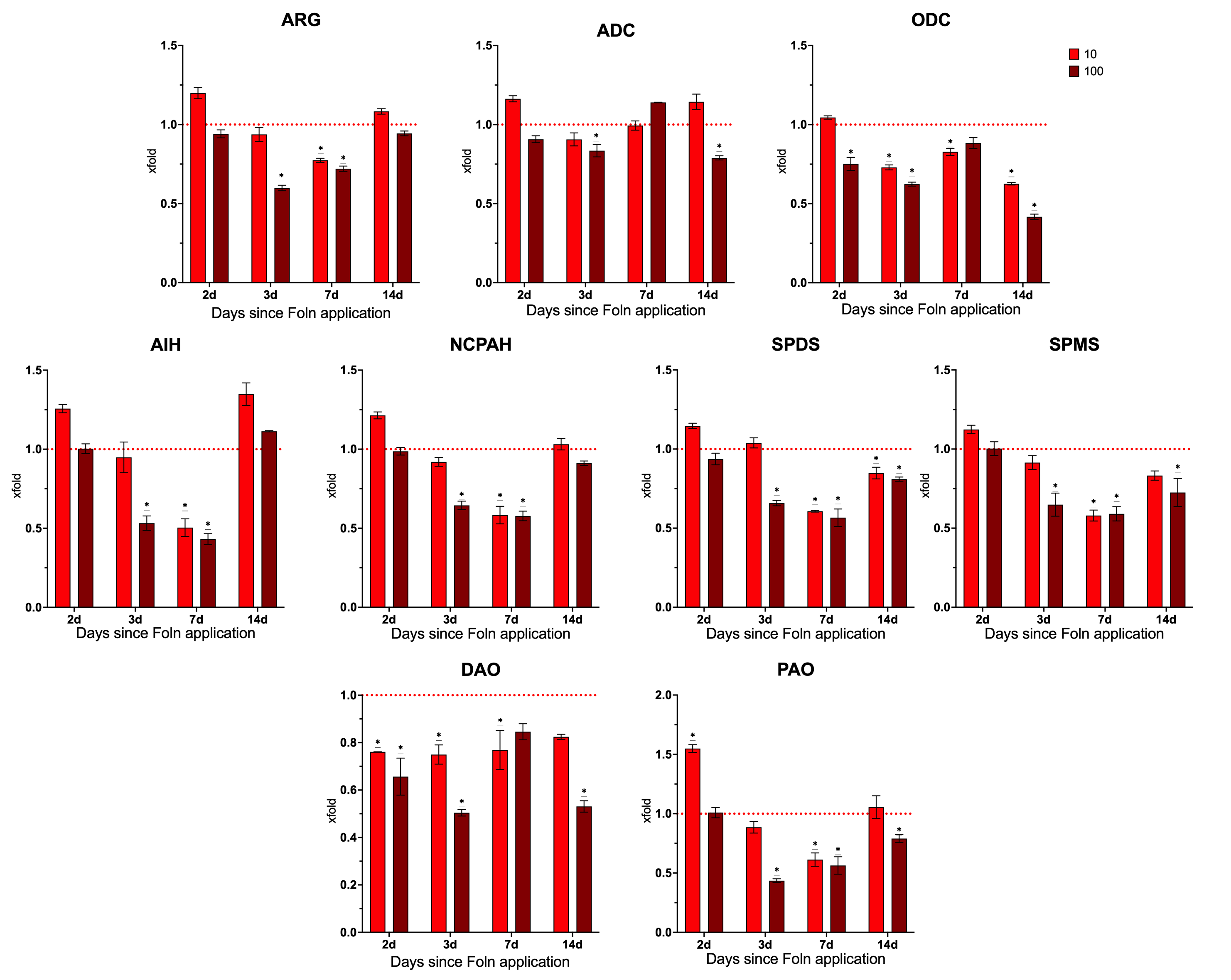


**Figure S8** Transcript level of polyamine metabolism genes in flax shoots after Spd treatment [10 and 100 mM] and Foln application. The data were obtained from real-time RT-PCR analysis. *Actin* was used as a reference gene and the transcript levels were normalized to the untreated infected plant. Bars represent the mean ± SD from three replicates. The significance of the differences was determined using two-way ANOVA with Tuckey post hoc test (*P<0,05) (*ARG arginase; ADC arginine decarboxylase; ODC ornithine decarboxylase; AIH agmatine iminohydrolase; NCPAH N-carbamoylputrescine amidohydrolase; SPDS spermidine synthase; SPMS spermine synthase; DAO diamine oxidase; PAO polyamine oxidase*).
